# Markov State Models Reveal a Two-Step Mechanism of miRNA Loading into the Human Argonaute Protein: Selective Binding followed by Structural Re-arrangement

**DOI:** 10.1371/journal.pcbi.1004404

**Published:** 2015-07-16

**Authors:** Hanlun Jiang, Fu Kit Sheong, Lizhe Zhu, Xin Gao, Julie Bernauer, Xuhui Huang

**Affiliations:** 1 Bioengineering Graduate Program, Division of Biomedical Engineering, The Hong Kong University of Science and Technology, Clear Water Bay, Kowloon, Hong Kong; 2 The HKUST Shenzhen Research Institute, Shenzhen, China; 3 Department of Chemistry, The Hong Kong University of Science and Technology, Clear Water Bay, Kowloon, Hong Kong; 4 Center of Systems Biology and Human Health, School of Science and Institute for Advance Study, The Hong Kong University of Science and Technology, Clear Water Bay, Kowloon, Hong Kong; 5 Computer, Electrical and Mathematical Sciences and Engineering Division, King Abdullah University of Science and Technology, Thuwal, Saudi Arabia; 6 Inria Saclay-Île de France, Bâtiment Alan Turing, Campus de l’École Polytechnique, Palaiseau, France; 7 Laboratoire d’Informatique de l’École Polytechnique (LIX), CNRS UMR 7161, École Polytechnique, Palaiseau, France; University of Missouri, UNITED STATES

## Abstract

Argonaute (Ago) proteins and microRNAs (miRNAs) are central components in RNA interference, which is a key cellular mechanism for sequence-specific gene silencing. Despite intensive studies, molecular mechanisms of how Ago recognizes miRNA remain largely elusive. In this study, we propose a two-step mechanism for this molecular recognition: selective binding followed by structural re-arrangement. Our model is based on the results of a combination of Markov State Models (MSMs), large-scale protein-RNA docking, and molecular dynamics (MD) simulations. Using MSMs, we identify an open state of apo human Ago-2 in fast equilibrium with partially open and closed states. Conformations in this open state are distinguished by their largely exposed binding grooves that can geometrically accommodate miRNA as indicated in our protein-RNA docking studies. miRNA may then selectively bind to these open conformations. Upon the initial binding, the complex may perform further structural re-arrangement as shown in our MD simulations and eventually reach the stable binary complex structure. Our results provide novel insights in Ago-miRNA recognition mechanisms and our methodology holds great potential to be widely applied in the studies of other important molecular recognition systems.

## Author Summary

In RNA interference, Argonaute proteins and microRNAs together form the functional core that regulates the gene expression with high sequence specificity. Elucidating the detailed mechanism of molecular recognition between Argonaute proteins and microRNAs is thus important not only for the fundamental understanding of RNA interference, but also for the further development of microRNA-based therapeutic application. In this work, we propose a two-step model to understand the mechanism of microRNA loading into human Argonaute-2: selective binding followed by structural re-arrangement. Our model is based on the results from a combined approach of molecular dynamics simulations, Markov State Models and protein-RNA docking. In particular, we identify a metastable open state of apo hAgo2 in rapid equilibrium with other states. Some of conformations in this open state have largely exposed RNA binding groove that can accommodate microRNA. We further show that the initial Argonaute-microRNA binding complex undergoes structural re-arrangement to reach stable binary crystal structure. These results provide novel insights into the underlying mechanism of Argonaute-microRNA recognition. In addition, our method is readily applicable to the investigation of other complex molecular recognition events such as protein-protein interactions and protein-ligand binding.

## Introduction

RNA interference (RNAi) is a key cellular mechanism involved in the regulation of gene expression and the defense against viruses[1,2]. As the central component of RNAi, the RNA induced silencing complex (RISC) mediates the sequence-specific recognition and inhibition of target mRNA[3,4]. At the core of the RISC, the Argonaute protein (Ago) in complex with the non-coding microRNAs (miRNAs) can selectively silence certain genes through specific binding to their mRNAs[5–12]. In human there are four Agos (hAgo1-4) and they together facilitate miRNA-based regulation of more than 30% of human genes[9,13]. While the miRNA libraries associated with different hAgos largely overlap, recent studies have identified exceptional miRNAs that bind specifically to only one hAgo[14–16]. Elucidating the molecular mechanisms of how Ago recognizes specific miRNAs is thus of vital importance not only for the fundamental understanding of RNAi but also for further development of miRNA-based therapeutics[17–19].

Protein-RNA recognition mechanisms are often interpreted via two popular models: conformational selection and induced fit[20,21]. In conformational selection, the RNA selectively binds to the bound conformation of the protein[22–24]. On the other hand, in induced fit, the RNA first binds to an unliganded protein conformation and further induces conformational changes of the protein to its bound form[25]. For example, the conformational selection model has been suggested in the recognition between human U2 snRNP auxiliary factor and polypyrimidine tract RNA[26]. On the other hand, the induced fit model plays a major role in the recognition between the *Xenopus* ribosomal protein L5 and the 5S rRNA[27]. Since both protein and RNA are large and flexible biopolymers, their recognition mechanisms often lie between the above two models[28–30]. Therefore, to dissect the protein-RNA recognition mechanisms, one needs to consider the chemical details of individual binding partners.

Despite intensive studies, the molecular mechanism of Ago-miRNA recognition still remains largely elusive. In the recently solved crystal structures of miRNAs in complex with eukaryotic Agos, miRNAs are deeply buried in the Ago binding groves, and this partially open bound conformation of Ago apparently prevents a direct loading of miRNA[31–34]. Therefore, a conformational selection model alone appears insufficient to describe the mechanisms of miRNA loading into Ago. Using a series of stopped-flow experiments, Deerberg *et al*. proposed a multi-step model for miRNA to load into human Argonaute-2 (hAgo2)[35]. However, to fill in their model with molecular details, further studies that elucidate the conformational dynamics of hAgo2 and miRNA are necessary.

An effective approach to investigate the possible role of the conformational selection in Ago-miRNA recognition is to examine if apo Ago can transiently reach a sufficiently open conformation to load miRNA. Molecular dynamics (MD) simulations offer a valuable tool to investigate the conformational dynamics of large biomolecules at atomic resolution. Previous MD studies at sub-microsecond timescales have demonstrated the impact of miRNA and double strand RNA on the conformational stability of the Ago complex[36–38]. However, solely using MD to model the complete loading process is extremely challenging due to the gap between the experimental timescale (at millisecond or longer) and that of all-atom MD simulations (typically at sub-microsecond). A feasible alternative approach is to address the above issue in two steps: to obtain the structural ensemble of the apo Ago protein with sufficient sampling followed by examining if any conformation in this ensemble can geometrically accommodate miRNA.

Markov state models (MSMs) hold great promise to describe the apo Ago conformational dynamics because it can bridge the timescale gap between MD simulations and experimental observations[39–50]. In an MSM, we coarse grain the conformational space into a number of metastable states, and simultaneously coarse grain the time in *Δt*. In this way, fast protein motions can be integrated out. When *Δt* is longer than the intra-state relaxation time, the model becomes Markovian. In other words, the probability for the system to visit a certain state at the next time step (*t+Δt*) is solely determined by its location at the current time step *t*. If the model is Markovian, we can model the long timescale dynamics using the first order master equation (see Eq (1)). In recent years, MSMs have been widely applied to study conformational dynamics of biopolymers[41,51–61].

Protein-RNA docking is a powerful tool to further examine if there exists apo Ago conformations that are open enough to accommodate miRNAs. The HADDOCK approach is particularly suitable for studying Ago-miRNA complexes because it is implemented with a flexible search algorithm that can benefit from the available information such as mutagenesis experiments and crystallographic contacts[62,63]. Performing HADDOCK simulations on apo Ago conformations identified by MSMs may provide an effective strategy to overcome the major limitation of protein-RNA docking, i.e. sufficiently considering the flexibility of both protein and RNA[64]. Furthermore, we can perform MD simulations initiated from docking poses to investigate subsequent conformational changes potentially induced by the binding.

In this work, we combine MSM with large-scale protein-RNA docking to elucidate the molecular mechanisms of miRNA loading into hAgo2. Our MSM identifies an open state of apo hAgo2 in fast equilibrium (at microsecond) with other metastable states. This open state has a characteristic structural feature: the miRNA binding groove is largely exposed to the solvent. Subsequent protein-RNA docking simulations confirm that a fraction of conformations in this state are sufficiently open to accommodate miRNAs. In further MD simulations initiated from the docking models, the complex undergoes structural re-arrangements to reach conformations closer to the crystal binary structure. Based on these observations, we propose a two-step mechanism for the recognition between miRNA and hAgo2: selective binding followed by structural re-arrangement.

## Results/Discussion

### Elucidating conformational dynamics of apo hAgo2 using MSMs

A 480-microstate MSM was initially constructed with the APM algorithm[65] from over 394,000 conformations based on the Euclidean distances of the two regions, namely the PAZ domain and two loops of the PIWI domain (the minor loop P601-P609 and the major loop V818-D838, see S1C Fig). These two regions were chosen since they are the most flexible moieties of apo hAgo2 dynamics in our MD simulations (see S1A Fig). Moreover, the MID domain displays the smallest conformational flexibility among all domains (see S1A Fig). For the N domain, even though it is more flexible than the MID domain, its location is away from the miRNA binding groove and thus may not directly affect the miRNA loading. Our model has been further validated by residence probability tests[45,54], in which the probability for the system to remain in a certain state predicted by the production MSM is in reasonable agreement with the probability obtained directly from MD trajectories (see S2B Fig).

Due to the large number of states, it is challenging to use our validated 480-microstate MSM for human appreciation of hAgo2’s conformational features. Therefore, we further lumped the microstates into seven metastable macrostates using the APM algorithm (see Methods for details). However, the residence probability tests show that the produced macrostate MSM predicts faster kinetics than the original MD simulations (see S2D Fig), even though the slowest implied timescales obtained from both MSMs are consistent (see S2C Fig). Therefore, all the quantitative properties reported in this study such as equilibrium populations and mean first passage times are obtained from the well-validated 480-microstate MSM.

**Fig 1:**
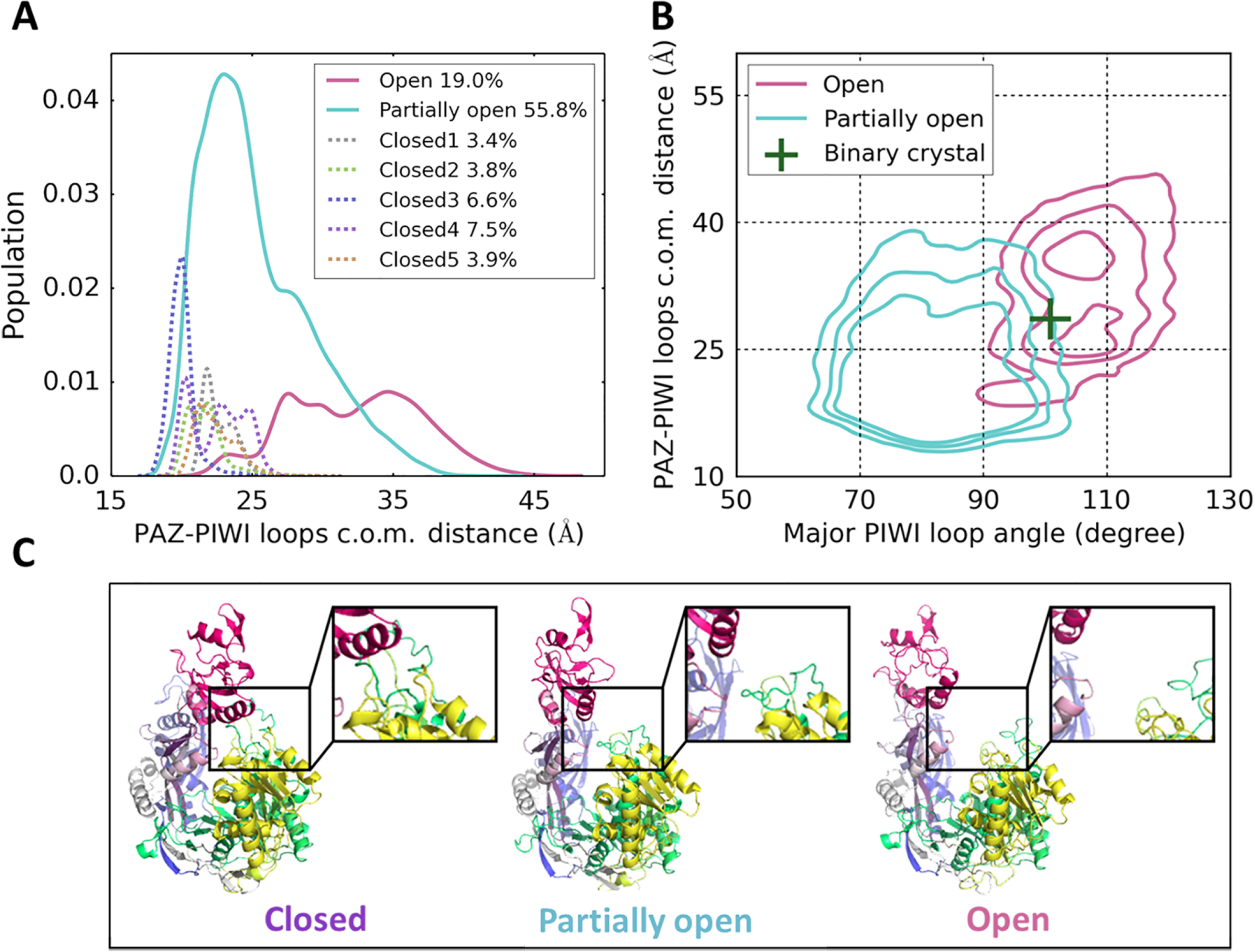
Visualization of the seven metastable macrostates obtained from MSM of apo hAgo2. (A) Distribution of the PAZ-PIWI loops center-of-mass (c.o.m.) distances for each macrostate. A large distance implies an open conformation. The equilibrium population of each macrostate is presented. (B) Projections of the open and partially open states onto PAZ-PIWI loops c.o.m. distance and the major PIWI loop angle (see S3 Fig for the angle definition). The green cross corresponds to the binary partially open crystal structure (missing residues modeled). (C) Representative structures of closed, partially open and open states. Enlarged view of the inter-domain region between PAZ (red) and PIWI (green) of each structure is presented in the inset panel (see representative structure for each macrostate in S4 Fig).

According to the degree of opening, the metastable conformational states of hAgo2 can be divided into three groups: an open state (19.0%), a partially open state (55.8%), and various closed states (see Fig 1A). Interestingly, the opening motion of hAgo2 can be effectively described by two PIWI loops and their interactions with the PAZ domain. The conformation of the PIWI loops in combination with their center-of-mass (c.o.m.) distance to the PAZ domain can not only separate the open state from the partially open state (see Fig 1B), but also distinguish five closed states (see S5 Fig). In these closed states, the PIWI domain can form direct interactions with the PAZ domain through multiple residues on both minor (e.g. D605) and major PIWI loops (e.g. E821, D823, E826, see S1B Fig).

Strikingly, the open state conformations can reach a widely open form with the c.o.m. distance between PAZ and PIWI loops as large as 46Å (see Fig 1A). In these conformations, the miRNA binding groove is already fully exposed to the solvent (see the right panel of Fig 1C), which strongly suggests that they may geometrically allow a direct loading of miRNA. In the next section, we will further examine this possibility via protein-RNA docking. Moreover, our MSM shows that the MFPTs between the open and closed state are only at an order of tens of microseconds (see S1 Table). These results indicate that the open conformations may be readily available to bind to miRNA through diffusion-controlled collision in the cellular environment. We also notice that these open conformations are even substantially more open than the binary partially open crystal structure (see the green cross in Fig 1B). These observations suggest that if miRNA can directly load into hAgo2’s open conformations, subsequent structural re-arrangements are necessary to reach the stable binary structure.

### Modeling miRNA loading into open hAgo2 conformations by protein-RNA docking and subsequent structural re-arrangements by MD simulations

To examine if the open conformations are able to accommodate miRNA, we performed large-scale protein-RNA docking using HADDOCK[62,63] on 150 selected open conformations of hAgo2. In particular, these conformations were selected from microstates with the c.o.m. distance between the PAZ domain and PIWI loops larger than 25Å (see Methods for details). For each docking simulation, we produced 50 hAgo2-miRNA structures. By computing their fraction of native contacts (***f***_***nat***_, see Eq (5)), we further show that the energy-based scoring function of HADDOCK can faithfully evaluate the quality of the docking structures. As shown in S6 Fig, the plot of the HADDOCK score against the fraction of native contact displays a nice funnel shape, indicating that the structure with the lowest HADDOCK scoring energy also preserves the most native contacts. We thus collected the lowest scoring energy structure from each of the 150 docking simulations for further examination.

**Fig 2:**
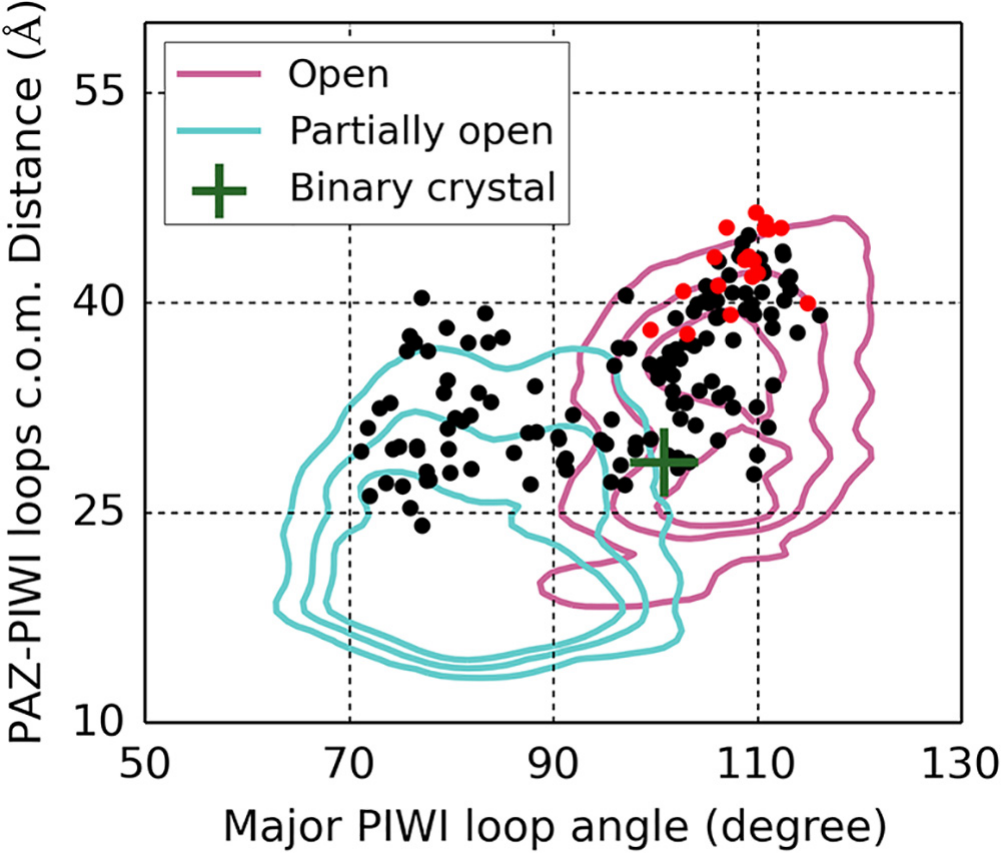
Projection of hAgo2-miRNA docking models built from the selected structures of open microstates. Red dots mark the successful docking models and black dots mark the unsuccessful ones. A successful docking model is a hAgo2-miRNA docking pose where at least two native contacts are preserved at each miRNA terminus.

Some of the lowest-energy docked structures have both 5’ and 3’ miRNA termini successfully anchored in the binding groove of hAgo2 (see red points in Fig 2), while most other structures have only one end of miRNA forming proper contacts with the protein (see black points in Fig 2). This observation indicates that not all hAgo2 conformations with a large distance between the PAZ and PIWI domains could geometrically accommodate miRNA. Other structural features such as the orientation of PIWI loops may also be necessary to define a sufficiently open binding groove of hAgo2. Indeed, in most successful docking models, the major PIWI loop forms an angle of over 100° with the rigid part of the PIWI domain (see Fig 2). Finally, we found that in our successfully docked structures, even though miRNA forms correct contacts with hAgo2, the Argonaute protein itself adopts a structurally more open conformation than that in its binary crystal (see S7 Fig). This indicates that structural re-arrangement is necessary after the initial binding of miRNA to hAgo2.

**Fig 3:**
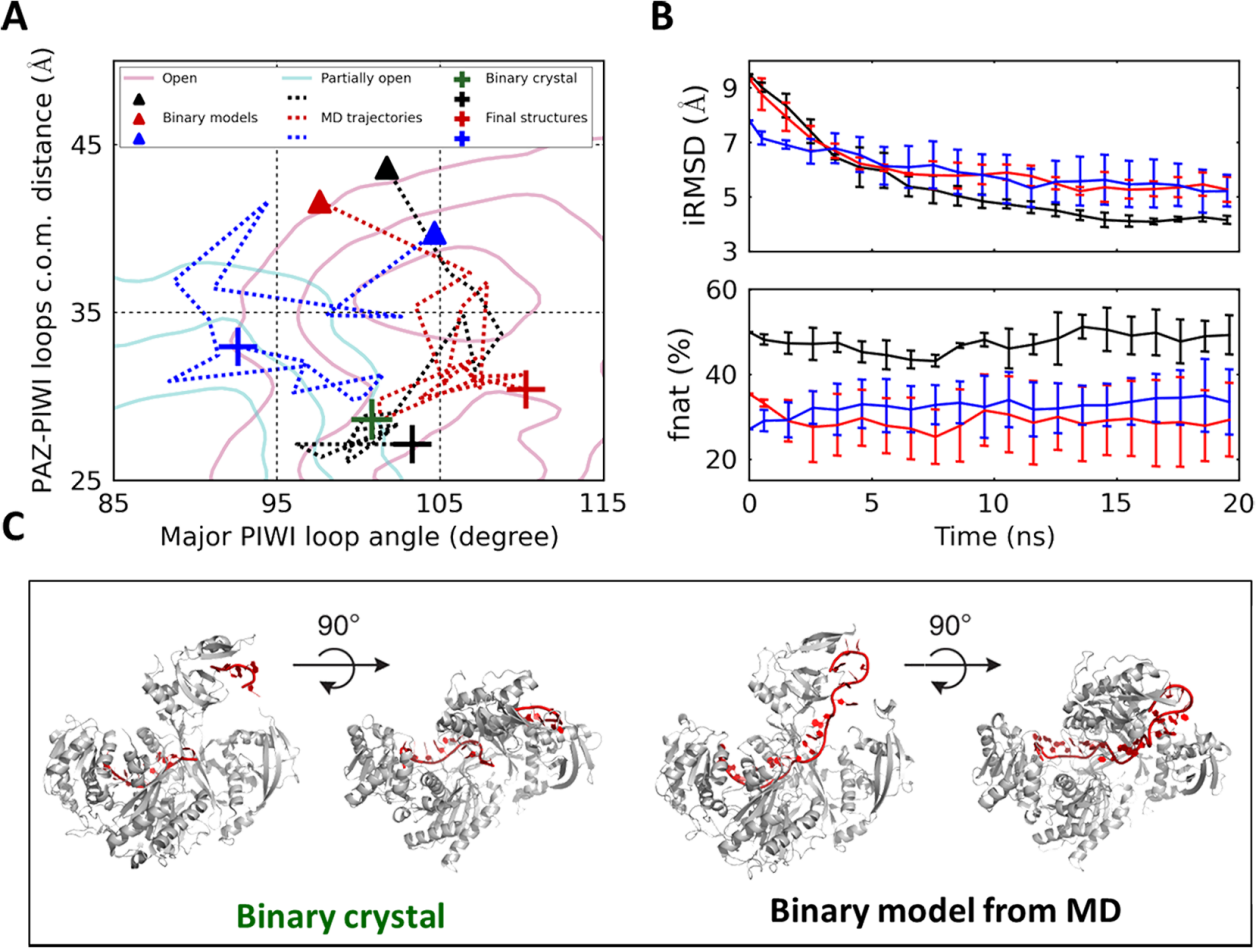
Binary docking models undergo substantial structural re-arrangement towards binary crystal during MD simulations. (A) Projections of MD trajectories of three representative successful docking models. The rationale behind the choice of representative docked conformations is simply to select conformations that contain different fractions of native contacts (i.e. the lowest: 27.1%; median: 35.4%, and highest: 50.0%). (B) Upper panel: interface-RMSD (iRMSD) of models against binary crystal structure. Lower panel: fraction of native contacts (f_nat_) between hAgo2 and miRNA of the models. (C) Structural comparison between the binary crystal structure (left) and a representative model from MD (right) with hAgo2 colored in grey and miRNA in red.

Initiating MD simulations from successfully docked structures, we observed substantial structural re-arrangement that brings the docked conformations closer to the binary crystal structure. In particular, we performed five independent 20-ns MD simulations from each of the three representative successfully docked conformations. MD simulations from these three representative conformations displayed decreased distance between the PAZ domain and PIWI loops, indicating that hAgo2 tends to close upon initial miRNA binding (see representative MD trajectories in Fig 3A and S8 Fig). We further show that such structural re-arrangement brings the hAgo2 conformation closer to the crystal structure (see the binding interface RMSDs in the upper panel of Fig 3B, the miRNA RMSDs in S9A Fig, and the hAgo2-miRNA salt bridges in S9C&S9D Fig) while still preserving protein-RNA native contacts (see lower panel of Fig 3B). In Fig 3C, we compare the binary crystal structure with a representative conformation after MD simulation, and these two structures display a high degree of structural similarity. Due to their limited lengths, our MD simulations could not fully reach the binary crystal structure, but they strongly suggest that protein-RNA interactions upon the initial miRNA loading may facilitate the further structural re-arrangement to reach the stable binary complex conformation.

### A model for the loading of miRNA into hAgo2

**Fig 4:**
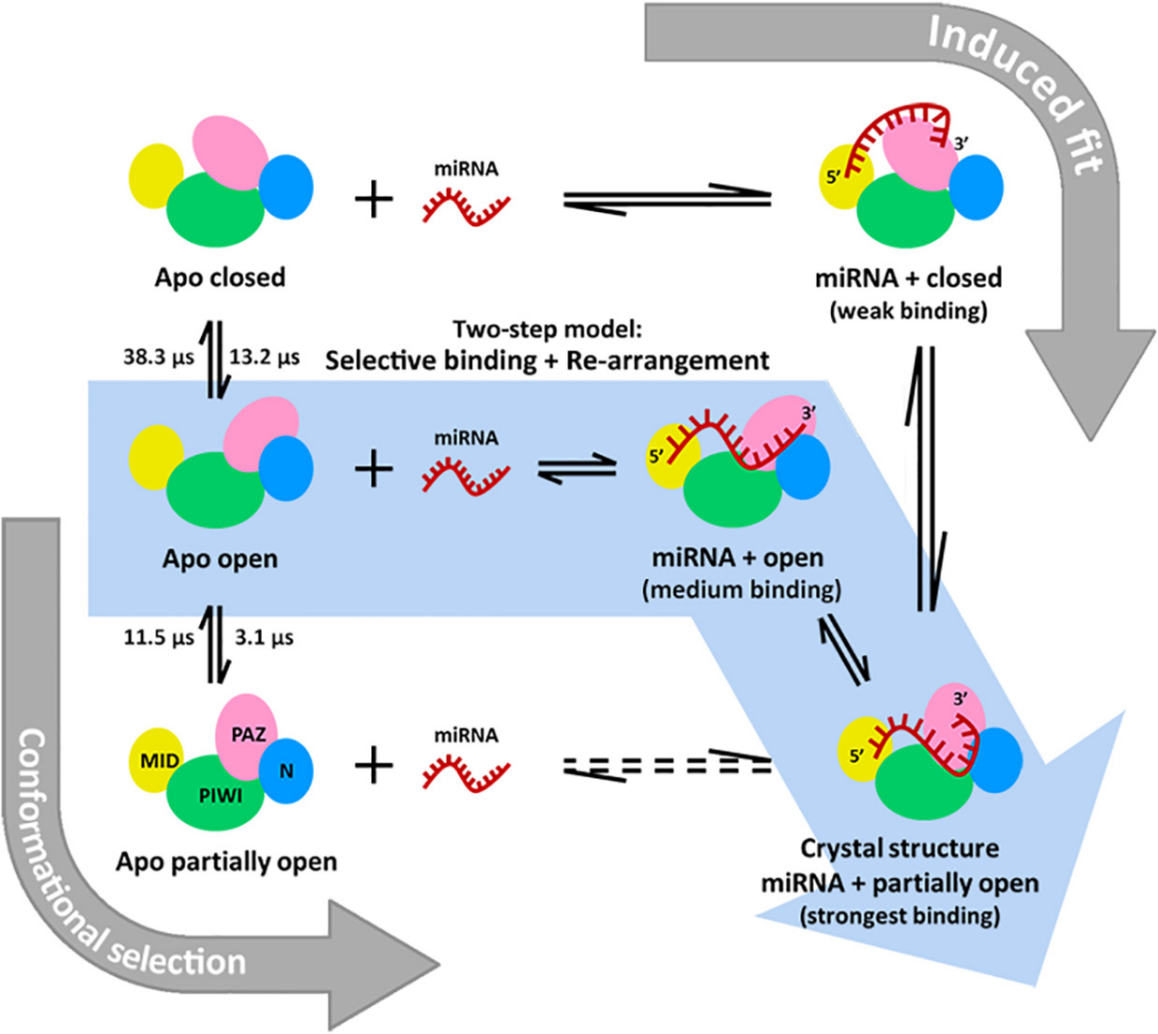
The proposed two-step model of miRNA loading into hAgo2: Selective binding followed by structural re-arrangement (highlighted by the cyan arrow). The induced fit mechanism (marked by the upper right grey arrow) and the conformational selection mechanism (marked by the lower left grey arrow) are also presented to compare with the two-step model. Average transition times between the closed states, the open state and the partially open states are computed from ten independent 10-ms synthetic trajectories generated by sampling the transition probability matrix of the 480-microstate MSM (see S10 Fig for additional details). The detailed transition pathways from the closed states to the open state can be found in S11 Fig.

Based on our results, we propose a two-step model of selective binding followed by structural re-arrangement model for the recognition mechanism between miRNA and hAgo2 (see Fig 4). In our model, the apo hAgo2 protein is very flexible and undergoes fast transitions among open, partially open and closed conformations. When miRNA encounters hAgo2, it can selectively bind to the open conformation. Upon the initial binding, the complex performs structural re-arrangement and eventually reaches the stable partially open binary complex conformation.

The proposed model is strongly supported by our MSMs built upon MD simulations of apo hAgo2, large-scale protein-RNA docking and MD simulations of the binary complex. In particular, our MSM identified a metastable open state of apo hAgo2 in fast equilibrium with other conformational states. Using protein-RNA docking, we demonstrate that some conformations in this open state can fully accommodate the miRNA. We further show that these open apo hAgo2 conformations can be accessed by miRNA since the rate of transition between the open and closed states (5 × 10^−2^ *Ms* ^−1^, see S1 Text for calculation details) is significantly faster than the collision rate between hAgo2 and miRNA under physiological conditions (1 × 10^−6^ *Ms* ^−1^, see S1 Text for calculation details). These results indicate that the initial hAgo2-miRNA recognition could involve selective binding of open hAgo2 by miRNA. Subsequent MD simulations initiated from docked conformations suggest that protein-RNA interactions may further induce conformational changes, and eventually this will drive the system to reach the stable binary conformation.

Notably, the selective binding step in our two-step mechanism has clear features of the conformational selection because the open hAgo2 state pre-exists in equilibrium with other conformational states, and miRNA selects to bind to this state with high binding affinity (see Fig 4). While in the original conformational selection mechanism the ligand usually selects the protein conformation with the highest affinity, our study suggests that miRNA cannot selectively bind to the partially open conformation of the highest affinity due to steric hindrance to the binding groove (see Fig 2 and S12 Fig). Instead, it chooses the open hAgo2 with the medium affinity as illustrated by our model (see Fig 4). This scenario, where the ligand selects a relatively high-affinity conformation but not the highest, has been previously observed in a number of molecular recognition system including ubiquitin binding[66] and LAO protein-arginine interactions[67], and sometimes regarded as the “extended conformational selection” model[21]. As pointed out by Boehr *et al*., conformational selection mechanism allows more promiscuous binding than induced fit[28]. Proteins that adopt this recognition mechanism may potentially bind to a larger pool of targets. Therefore, our model may help understand the ability of hAgo2 to bind to miRNAs of different lengths and sequences.

Our simulations complement previous experiments and fill in molecular details for the understanding of hAgo2-miRNA recognition mechanisms. Deerberg *et al*. showed that three rate constants could nicely fit the kinetics of RNA binding to hAgo2 from their stopped-flow experiments[35]. Based on these observations, they proposed a 3-step model: formation of hAgo2-RNA collision complex, anchoring of the 5’ terminus of the RNA and anchoring of the 3’ terminus[35]. Interestingly, the initial hAgo2-RNA collision rate was orders of magnitude faster than the subsequent anchoring of RNA termini in their model. Based on our simulations, we suggest that the initial hAgo2-miRNA collision observed in stop-flow experiments could contain the preferential binding of miRNA to an open conformation of hAgo2. The structural re-arrangement of the complex after the initial loading could include anchoring of miRNA termini in a sequential order. Since a significantly larger number of contacts are formed between hAgo2 and 5’ miRNA (43 contacts) compared to the 3’ miRNA (only 5 contacts) in the crystal structure, we speculate that 5’ miRNA may successfully anchor to the binding groove of hAgo2 first. To further investigate this issue, one needs to explicitly simulate the collision and binding between flexible miRNA and hAgo2 in solution. Considering the experimental timescale (at ~100 seconds[35]), this will be extremely challenging for all-atom MD simulations. We also note that the structural re-arrangement step in our proposed model can also be regarded as an induced fit mechanism. Indeed, our results do not rule out the possibility that induced fit mechanism plays a role during the initial hAgo2-miRNA recognition. However, it may be difficult for the induced fit model alone to explain multiple distinct timescales observed in the stop-flowed experiments.

**Fig 5:**
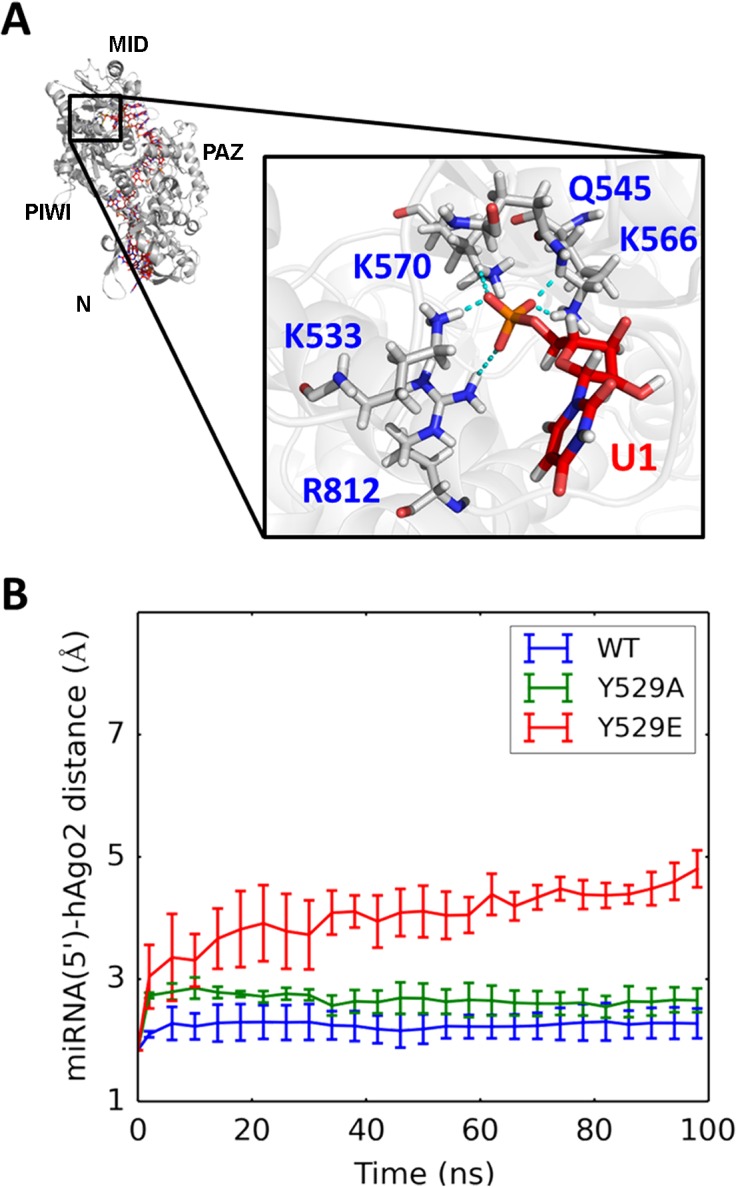
Mutations in the binding pocket for miRNA(5') show different effects on hAgo2-miRNA binding. (A) miRNA(5’)-hAgo2 distance is defined as the minimum distance between U1 of miRNA and its binding pocket in hAgo2 (K533, Q545, K566, K570 and R812). The increase of the distance is an indicator of the weaker hAgo2-miRNA interactions. (B) Time traces of miRNA(5’)-hAgo2 distance in wild type (WT) hAgo2 (blue), the Y529A mutant (green) and the Y529E mutant (red). Error bars were computed from five independent MD simulations.

**Fig 6:**
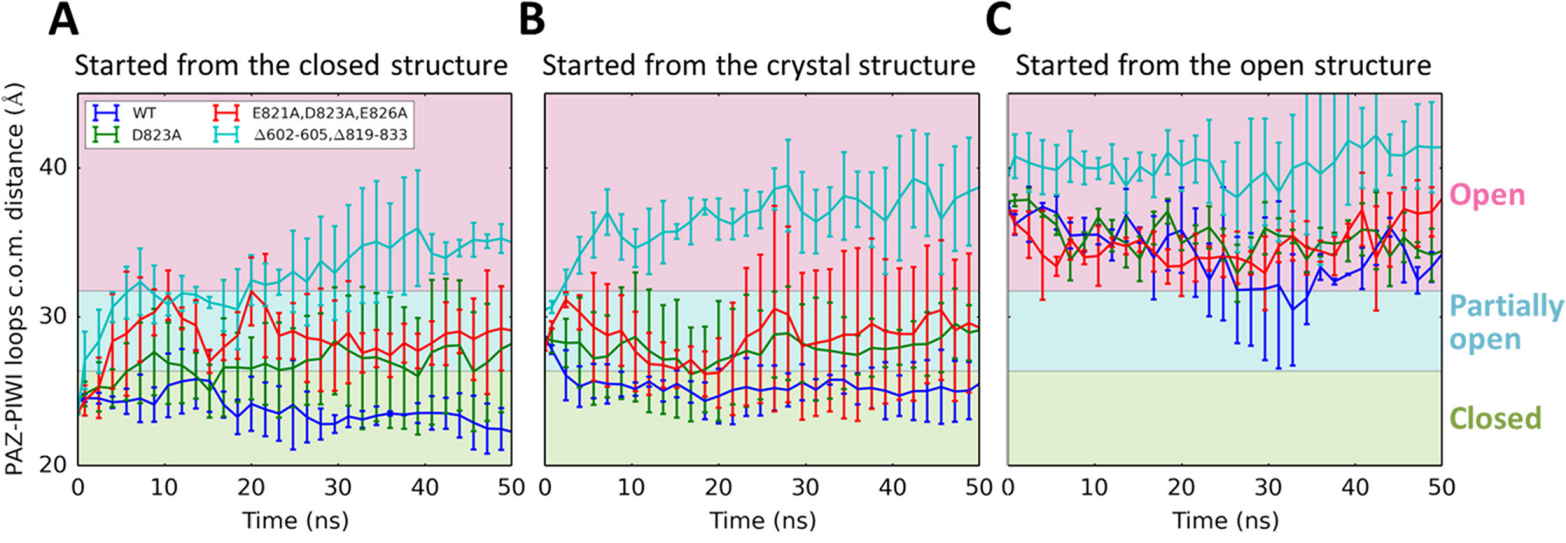
Mutations in PIWI loops destabilize the closed conformation and accelerate the closed-to-open transitions. Three mutants were generated: D823A (green), E821A-D823A-E826A (red) and ∆602–605∆819–833 (a deletion mutant where both PIWI loops are truncated, cyan). MD simulations of WT hAgo2 (blue) and the mutants were initiated from three conformations: (A) a closed conformation, (B) a partially open conformation extracted from the binary hAgo2-miRNA crystal structure (PDB ID: 4F3T) and (C) an open conformation. Time traces of the c.o.m. distance between the PAZ domain and PIWI loops of the WT hAgo2 and the three mutants are displayed. Error bars were computed from five independent MD simulations.

Our simulations also generate predictions that can be tested by experiments. For example, two mutants (Y529A and Y529E) were designed with modified binding pocket for the 5’ terminus of miRNA. MD simulations of these two mutants display distinct features: Y529A mutant largely preserves the interactions between miRNA and hAgo2, while Y529E mutant significantly disrupts the protein-RNA interactions, resulting in substantial increase in the distance between miRNA and its binding pocket (see Fig 5). We note that even though one may need much longer simulations to thoroughly examine the stability of the hAgo2-miRNA complex, within the timescale we simulate (at 100ns), we already clearly see that the Y529E mutant forms a less stable complex with miRNA compared to the WT and the Y529A mutant. These predictions are consistent with previous experiments showing that miRNA can bind to the Y529A mutant, but not the Y529E mutant[68,69]. More interestingly, since our MSM shows that interactions between the PIWI loops and the PAZ domain can stabilize the apo hAgo2 in the closed state, we designed three modifications on PIWI loops (single mutant D823A, triple mutant E821A-D823A-E826A and deletion mutant ΔP602-D605ΔD819-Q833) that may cause hAgo2 to favor more the open state. Initiated from a closed conformation, the WT hAgo2 stays in the closed state throughout the 50-ns MD simulations (see Fig 6A). This is expected as our MSM predicts that it may take tens of microseconds for the WT hAgo2 to diffuse out of this state (see S10 Fig). However, the deletion mutant (Δ602–605Δ819–833) undergoes fast conformational changes and reaches the open conformation within 20ns (see Fig 6A). The other two mutants (D823A and E821A-D823A-E826A) also quickly escape from the closed conformational state and reach partially open conformations (see Fig 6A). As shown in Fig 6B, the mutant simulations initiated from a partially open conformation are mostly consistent with those started from the closed conformation. Since this initial hAgo2 conformation is extracted from a partially open crystal structure of hAgo2-miRNA complex, the removal of miRNA may leave space and further lead to the collapse of the hAgo2 to reach the closed state in the WT MD simulations. As controls, we have also performed MD simulations of the WT hAgo2 and the mutants initiated from an open conformation (see Fig 6C). As expected, all the systems remain in the open conformation throughout the MD simulations. Taken together, the above observations suggest that the PIWI loops mutations are likely to accelerate the transition from the closed to the open conformation by destabilizing the closed state. We hence predict that these mutations could facilitate the initial binding of miRNA by modulating hAgo2’s conformational dynamics to make its open state more accessible, even though these mutants are located far from the protein-RNA binding interface. We note that the interaction between PAZ and the PIWI loops not only regulates the molecular recognition between hAgo2 and miRNA, but could also play a role during mRNA recognition by hAgo2-miRNA complex (see S2 Text for detailed discussions).

In conclusion, by combining MSMs, large-scale protein-RNA docking, and MD simulations, we propose a two-step mechanism for the molecular recognition between miRNA and hAgo2: selective binding followed by structural re-arrangement. Using MSMs, we identified an open state of apo hAgo2 in rapid equilibrium with partially open and closed states of this enzyme. Conformations in this open state can contain a widely open binding groove, which are shown to be able to accommodate miRNA in our protein-RNA docking simulations. We thus suggest that the initial binding of miRNA to hAgo2 adopts a selective binding mechanism. This initial binding complex may undergo further structural re-arrangement to finally reach the stable binary complex structure. Our model is consistent with previous experimental results, and fills in more details at molecular level. We have also made predictions that can be tested experimentally. Our studies provide novel insights in fundamental mechanisms of how the Argonaute protein recognizes miRNA, which plays an important role in the gene regulation by RNA interference. The methodology here also holds great promise to be widely applied to investigate molecular recognition mechanism of other biological event, such as protein-protein interactions and protein-ligand binding.

## Methods

### MD simulation setup

The initial apo hAgo2 conformation was taken from the crystal structure of hAgo2 in complex with miR-20a (PDB ID: 4F3T)[32], and the missing residues were added using MODELLER[70–73]. The apo hAgo2 protein was solvated in a dodecahedron box containing 42,847 SPC water molecules[74] with 129 Na^+^ ions and 159 Cl^-^ ions to neutralize the charge. All the MD simulations were performed using the GROMACS 4.5.4 package[75] and the Amber99SB-ILDN force field[76]. Long-range electrostatic interactions were treated with the Particle-Mesh Ewald method[77]. Both short-range electrostatic interactions and van der Waals interactions used a cutoff of 10Å. All bonds were constrained by the LINCS algorithm[78]. Velocity-rescaling thermostat[79] and the Parrinello-Rahman barostat [80] were used for temperature and pressure coupling respectively. The system was first energy minimized with the steepest descent algorithm and equilibrated for 1ns under NPT ensemble (T = 310K, P = 1atm) with the positions of all the heavy atoms restrained. Next, we performed a 5-ns NPT simulation (T = 310K, P = 1atm) to further equilibrate the system. Finally, all the production MD simulations were performed under NVT ensemble at 310K.

After the equilibration, we first performed six independent MD simulations ranging from 50 to 100ns which summed up to ~400ns. We then divided the conformations obtained from these six MD simulations (saved every 20ps with a total of ~20,000 conformations) into 20 clusters using the K-centers algorithm[81]. We randomly selected one conformation from each cluster and initiated a second round of 150-ns MD simulations with one simulation from each of these 20 conformations. Next, we extracted ~170,000 conformations from the first two rounds of MD simulations and built an MSM containing 553 microstates that were further lumped into 9 macrostates. However, the 553-microstate MSM predicted kinetics inconsistent with the original MD simulations, indicating the necessity of additional sampling (see S13 Fig). We thus seeded the third round of MD simulations from conformations taken from each of the 9 macrostate in the initial MSM with the number proportional to their predicted equilibrium populations. In this round, we performed 30 150-ns MD simulations with a saving interval of 20ps. Combining MD simulations from all three rounds, we obtained a dataset containing over 394,000 conformations extracted from ~8μs MD simulations.

### Construction of MSM

When constructing MSMs, one divides the conformational space into a group of metastable states, and coarse-grains time into a discrete interval *Δt*. If the model is Markovian, an MSM can predict the long-timescale dynamics via the first-order master equation:

$$p\left(n\Delta t\right)=T^{n}\left(\Delta t\right)p\left(0\right),$$ (1)

where *p(nΔt)* is the vector describing state population and *T(Δt)* is the transition probability matrix of the lag time *Δt*.

To construct MSMs from MD simulations, we applied our recently developed Automatic state Partitioning for Multi-body systems (APM) algorithm[65]. The main insight of the APM algorithm is to take into account the kinetic information when performing the geometric clustering, which is often based on the RMSD between pairs of MD conformations. This is achieved by splitting the conformations using a divide-and-conquer scheme until all the resulting microstates has a common maximum residence time *t*_*0*_. The residence time of a certain microstate *i* is measured by relaxation of the transition probability out of this state or escaping probability *P(i*, *t)*:

$$P\left(i,t\right)=1-\frac{\sum_{j=1}^{n}\sum_{k=0}^{\left(T_{j}-t\right)/s}\delta \left(m_{j}\left(ks\right)-i\right)\delta \left(m_{j}\left(ks+t\right)-i\right)}{\sum_{j=1}^{n}\sum_{k=0}^{\left(T_{j}-t\right)/s}\delta \left(m_{j}\left(ks\right)-i\right)},$$ (2)

where *m*_*j*_ *(t)* indicates which microstate the system is in at time *t*. *T*_*j*_ is the length of the *j*-th MD trajectory with conformations stored at a time interval of *s*. We use the lifetime (*t*_*i*_) of microstate *i*, estimated by *P(i*, *t*_*i*_ *) = 1-1/e*, as the indicator of its residence time. If *t*_*i*_ *< t*_*0*_ (*t*_*0*_ is the predetermined maximum residence time), the system can relax out of a microstate within *t*_*0*_.

The detailed procedure of the APM algorithm is as follows[65] (see S14 Fig): (1) Perform a geometric clustering using the K-centers algorithm[81] to divide MD conformations into two microstates. (2) Examine the residence time of each microstate. For microstate *i*, if *t*_*i*_ *≥ t*_*0*,_ we further split it until *t*_*i*_ *< t*_*0*_. We can then obtain a set of microstates with an upper limit on the residence time. (3) Lump kinetically related microstates into metastable macrostates using spectral clustering[82]. (4) Perform multiple iterations of re-splitting and re-lumping to remove potential internal free energy barriers within microstates.

To apply the APM algorithm, we chose the maximum residence time *t*_*0*_ = 20ns, and this resulted in 480 microstates that were subsequently lumped into seven macrostates by spectral clustering[82]. To compute the distance between each pair of conformations, we first aligned all MD conformations to the crystal structure against its PIWI Cα atoms, and then computed Euclidean distances based on Cα atoms of the PIWI loops and every 3rd Cα atoms of L1L2 linking regions in the PAZ domain. In this way, both PIWI loops and PAZ-L1L2 regions, the two critical components that determine the magnitude of opening of hAgo2, have relatively equal contributions to the pair-wise distance.

### Validation of MSM

The implied timescale *τ*_*t*_ is defined by the following equation:

$$\tau_{t}=-\frac{\tau }{\ln\lambda_{k}},$$ (3)

where *λ*_*k*_ is the *k*-th largest eigenvalue of the transition probability matrix of *τ*.

As shown in S2A Fig, the implied timescale plots of the 480-microstate MSM level off at around 20ns, indicating that the model is Markovian at this or longer lag time. In our production MSM, we selected 20ns as the lag time. To further validate our model, we also performed residence probability tests[45,54]. During these tests, we compared the MSM-predicted probability of the hAgo2 staying in a certain microstate with the probability derived by counting the transitions in MD trajectories. As shown in S2B Fig, the two probabilities agree well with each other, suggesting that our MSM captured the kinetics of apo hAgo2 sufficiently well.

We further lumped the 480 microstates into seven macrostates because of the presence of a clear gap between 6^th^ and 7^th^ slowest timescales in the implied timescale plots of the microstate MSM. The implied timescale plot of the 7-macrostate MSM was congruent with that of microstate MSM (see S2C Fig). However, this model predicted faster dynamics than that shown by MD trajectories during the residence probability tests (see S2D Fig). Therefore, the 7-macrostate MSM model was used for visualization of the state decomposition and the 480-microstate MSM was used for calculating quantitative properties reported in this study. For example, we summed over the equilibrium populations of all the microstates that belong to a certain macrostate to obtain their populations.

### Mean first passage time (MFPT)

MFPT was used to estimate the dynamics between the open and the closed states. It is defined as the average time it takes for the system to visit the final state *f* from the initial state *i*. We computed MFPT between the open and the closed states by solving the following equation:

$$X_{if}=\Sigma_{j}T{\left(\tau \right)}_{ij}\left(\tau +X_{jf}\right),$$ (4)

where *T*(*τ*)_*ij*_ is the transition probability from state *i* to state *j*, *τ* is the lag time with *X*_*ff*_ = 0.

To compute the MFPT between a pair of macrostates from the 480-microstate MSM, we set MFPTs to be zero for all the microstates that belong to the final macrostate. We then computed MFPTs starting from each of the microstates that belong to the initial macrostate, and performed a weighted average over these MFPTs according to the normalized equilibrium populations of these microstates predicted by the 480-microstate MSM. We also performed a cross validation of the MFPTs calculated from our MSM by evenly dividing the dataset into two non-overlapping sub-datasets. MFPTs predicted from MSMs built from these two sub-datasets are in reasonable agreement with each other, and are also consistent with the MFPTs reported using the whole dataset (see S15 Fig).

### Protein-RNA docking with HADDOCK

Protein-RNA docking simulations were performed with HADDOCK 2.1[62,63]. Since the RNA binding groove is completely blocked or largely hindered by the PIWI loops in the closed or partially open hAgo2 conformations (see S4 Fig for representative conformations), these conformations are unlikely to allow the direct miRNA binding. Therefore, we selected hAgo2 conformations that are as open as possible (with PAZ-PIWI loops c.o.m. distance > 25Å) and obtained 15 microstates. From each of these 15 microstates, we randomly selected 10 conformations for docking. The input miRNA structure was modeled using ModeRNA[83] based on miRNA fragments in the crystal structure (PDB ID: 4F3T).

We defined both Ambiguous Interaction Restraints (AIR) and unambiguous distance restraints to drive the docking simulations. For AIR, the “active” protein residues were defined according to the hAgo2-miRNA interactions suggested by Elkayam *et al*.[32]: R179, S180, A221, T222, H271, K278, R280, F294, Y311, H316, R351, G524, K525, T526, Y529, K533, Q545, C546, Q548, N551, S561, K566, K570, D597, R635, D669, R710, Q757, R761, R792, S798, Y804, H807, and R812. All RNA nucleotides were set to be “active”. We also imposed unambiguous distance restraints to guide the docking simulations (see S2 Table).

The inter- and intra-molecular interactions were calculated by CNS 1.3[84,85] with PARALLHDG5.4[86,87] and the OPLS-AA force field[88] applied to protein and miRNA respectively. For each docking simulation, topologies and coordinates of hAgo2 and miRNA were generated from input structures using CNS. The two molecules were initially separated 25Å away before the randomization and rigid body energy minimization. 200 docking poses were generated after the rigid body docking and ranked by the HADDOCK score, a weighted sum of various energy terms (electrostatic, van der Waals, desolvation, ambiguous interaction restraints and buried surface area). The 50 best-scoring poses were subsequently used for semi-flexible simulated annealing and the final solvated refinement.

Besides the HADDOCK score, we also employed an adapted definition of the fraction of native contacts (f_nat_)[89] to examine the quality of docking structures. The fraction of native contacts is defined as:

$$f_{nat}=\frac{q_{pose}}{q_{crystal}},$$ (5)

where *q*_*pose*_ and *q*_*crystal*_ denote the numbers of protein-miRNA contacts (distance < 4Å) observed in the docking pose and in the crystal structure, respectively.

In total we collected 48 hAgo2-miRNA contacts from the crystal structure (see S3 Table).

Since HADDOCK cannot fully consider the conformational dynamics of hAgo2-miRNA complex upon the initial binding, we note that subsequent MD simulations are required to refine the docking poses.

## Supporting Information

S1 Fig (A) The residue Root Mean Square Fluctuations (RMSF) of apo hAgo2. The PAZ domain and the major loop of the PIWI domain are the most flexible hAgo2 moieties. (B) Frequency of PIWI loops residues participating in PIWI-PAZ hydrogen bonding. (C) Domains of hAgo2. Major and minor loops of PIWI are highlighted in cyan and orange respectively. The region where the PIWI loops interact with PAZ is enlarged in the right inset panel.
(TIF)

S2 Fig Validation of the 480-microstate MSM and 7-macrostate MSM. (A) Implied timescale of microstates. (B) Residence probability tests of randomly selected representative microstates. The red curves indicate probability obtained from MD simulations and the black curves indicate probability predicted by MSM. (C) Implied timescale of macrostates. (D) Residence probability tests of macrostates. The coloring scheme is the same with (B).
(TIF)

S3 Fig Definition of the major PIWI loop angle. This angle describes the position of the Cα atom in D823, a residue on major PIWI loop which frequently participates in the interaction with PAZ. It thereby indicates the conformation of the major PIWI loop. When the angle is small, the major loop flips towards PAZ and easily forms hydrogen bonding network; when this angle is large, the major loop flips away from PAZ and diminishes PAZ-PIWI loops interactions. MID domain is not shown for a clear view on the angle.
(TIF)

S4 Fig Representative structures of macrostates. The structures of the PAZ domain and the PIWI loops are highlighted in the inset panels. Their equilibrium populations predicted from our MSM are also shown.
(TIF)

S5 Fig Characterization of five closed MSM macrostates via principal component analysis (PCA) of PIWI loops Cα atoms. (A) The 2^nd^ and 4^th^ eigenvectors from the PCA. PIWI loops are colored in blue and red arrows mark the direction of the corresponding eigenvector. (B) Projection of the five closed states on the plane of the 2^nd^ and 4^th^ eigenvectors. The 2^nd^ and 4^th^ eigenvectors were chosen for projection because they can clearly separate the closed macrostates from each other.
(TIF)

S6 Fig Evaluation of binary hAgo2-miRNA models from protein-RNA docking. (A) Projection of 50 binary models from one protein-RNA docking simulation on their HADDOCK scores (vertical axis) and the fraction of native contacts preserved of the models (horizontal axis). (B) Structures of the worst (top) and the best (bottom) docking binary models from the 50 models presented in (A).
(TIF)

S7 Fig Structural comparison between the binary crystal structure (left) and one of the successful binary docking model (right) with hAgo2 colored in grey and miRNA in red. (TIF)

S8 Fig Time traces of PAZ-PIWI loops c.o.m. distances of three successfully docked hAgo2-miRNA models during MD simulations. Each curve shows the average and the fluctuation of three individual trajectories from the same docked structure. The green line marks the PAZ-PIWI loops c.o.m. distance of the partially open crystal hAgo2 structure.
(TIF)

S9 Fig (A) RMSD curves of miRNA against its conformation in the binary crystal structure as a function of time. The results of three MD simulations from a successfully docked hAgo2-miRNA structure are shown. (B) Backbone Root Mean Square Fluctuations (RMSFs) of individual nucleotide in the miRNA. (C) Fraction of the native hAgo2-miRNA salt bridges formed as a function of simulation time. Results from the same three MD simulations as in (A) are shown. Native salt bridges are defined as those found in the binary hAgo2-miRNA crystal structure. (D) Representative snapshots from a MD simulation indicate the formation of the salt bridges: R68-U12, R69-G14 and K278-G14. The snapshots are selected from the MD simulation shown in black in part (A).
(TIF)

S10 Fig Long trajectories predicted from the MSM show that the apo hAgo2 can make transitions between the closed and open conformations at tens of microseconds. (A) PAZ-PIWI loops c.o.m. distance as a function of time in an MSM-predicted 60μs segment of trajectory. This trajectory is initiated from a closed conformation (Closed2 state). To obtain this trajectory, we sample the transition probability matrix of the validated 480-microstate MSM. In each time-step (20ns as the lag time in our MSM), the next microstate that the system will visit is determined by a randomly selected microstate according to the corresponding transition probability in the MSM. A random conformation from this microstate is then selected to compute the PAZ-PIWI loops c.o.m. distance. (B) The same as (A) except that the trajectory is started from the open conformation. (C) The same as (A) except that the macrostate ID as a function of time is displayed. To obtain the macrostate ID, we simply map the microstate the system visits to its corresponding macrostate. (D) The same as (B) except that the macrostate ID as a function of time is displayed.
(TIF)

S11 Fig Superimposition of the ten flux pathways from the closed states to the open state. The flux is calculated using a greedy backtracking algorithm from the validated 480-microstate MSM[59] and (Weinan *et al*, *J*. *Stat*. *Phy*., 2006, 503–523). By applying the Transition Path Theory (Weinan *et al*, *J*. *Stat*. *Phy*., 2006, 503–523) to the 480-microstate MSM, we identify over one thousand pathways based on the microstates that can be further combined into ten pathways from the closed macrostates to the open macrostate. For each state a representative structure is displayed together with the MSM-predicted equilibrium population. The size of the arrows is proportional to the interstate flux.
(TIF)

S12 Fig The partially open conformation of hAgo2 in the co-crystal structure (PDB ID:4F3T) cannot accommodate miRNA. (A) Comparison between the preserved native contacts of the best-scoring docked crystal hAgo2 and the average native contacts of all the successfully docked open conformations of hAgo2. (B) Structural comparison between the best scoring docking pose and the crystal hAgo2-miRNA complex. miRNA in the docked conformation locates outside of the RNA binding groove of hAgo2.
(TIF)

S13 Fig The preliminary 553-microstate MSM based on apo hAgo2 conformations from the first two rounds of MD simulations predicts kinetics inconsistent with MD simulations. Residence probability tests of randomly selected microstates are presented. The red curves indicate probability obtained from MD simulations and the black curves indicate probability predicted by MSM.
(TIF)

S14 Fig A schematic representation of the APM algorithm. In the first step, a recursive geometric clustering is performed to divide MD conformations into microstates, until the residence times of all the microstates are below the same upper threshold. In the second step, kinetically related microstates are lumped into macrostates. Finally, multiple iterations of re-splitting and re-lumping are performed to optimize the state decomposition.
(TIF)

S15 Fig Comparison of kinetics (MFPTs) predicted by MSM based on 50% of MD simulations and the MSM based on all of the simulations. The two 50% datasets are non-overlapping subsets of the 100% dataset. The five closed states are combined and considered as one state during the calculation of MFPTs. The error bars are generated by bootstrapping N trajectories from MD dataset (N being the number of trajectories in the dataset) with replacement for N times.
(TIF)

S1 Table Mean first passage time (MFPT) among MSM states. Each number indicates the MFPT from the row macrostate to the column macrostate. The uncertainties were obtained from bootstrapping the MD dataset (containing 56 trajectories) with replacement for 56 times. The unit of time is μs.
(PDF)

S2 Table Unambiguous distance restraints for HADDOCK simulations. The restraints were selected from the closest contacts between hAgo2 and terminal nucleotides of miRNA in the crystal structure (PDB ID: 4F3T).
(PDF)

S3 Table hAgo2-miRNA native contacts from the crystal structure. All the hAgo2-miRNA distances within 4Å are recorded.
(PDF)

S1 Text Comparison between apo hAgo2 dynamics and kinetics of hAgo2-miRNA collision. (PDF)

S2 Text PAZ-PIWI loops interaction could enhance the fidelity of mRNA target recognition. (PDF)
