# Supplementary figures and images for: Markov State Models Reveal a Two-Step Mechanism of miRNA Loading into the Human Argonaute Protein: Selective Binding followed by Structural Re-arrangement
Source: PLoS Comput Biol. 2015 Jul 16;11(7):e1004404. doi: 10.1371/journal.pcbi.1004404 (PMC4504477; doi:10.1371/journal.pcbi.1004404)

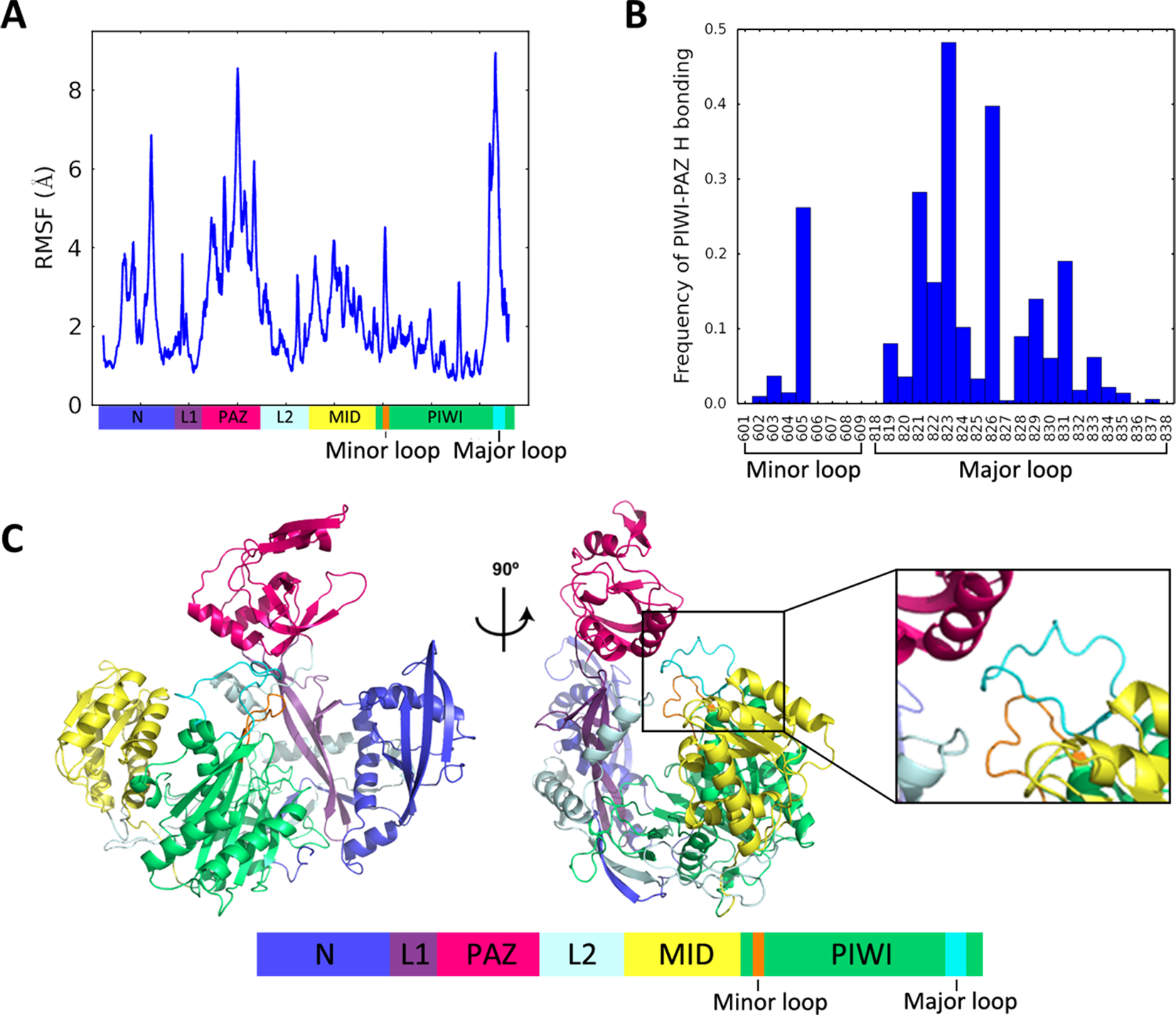

Supplement: S1 Fig — The PAZ domain and the major loop of the PIWI domain are the most flexible hAgo2 moieties. (B) Frequency of PIWI loops residues participating in PIWI-PAZ hydrogen bonding. (C) Domains of hAgo2. Major and minor loops of PIWI are highlighted in cyan and orange respectively. The region where the PIWI loops interact with PAZ is enlarged in the right inset panel. (TIF) [file pcbi.1004404.s001.tif]

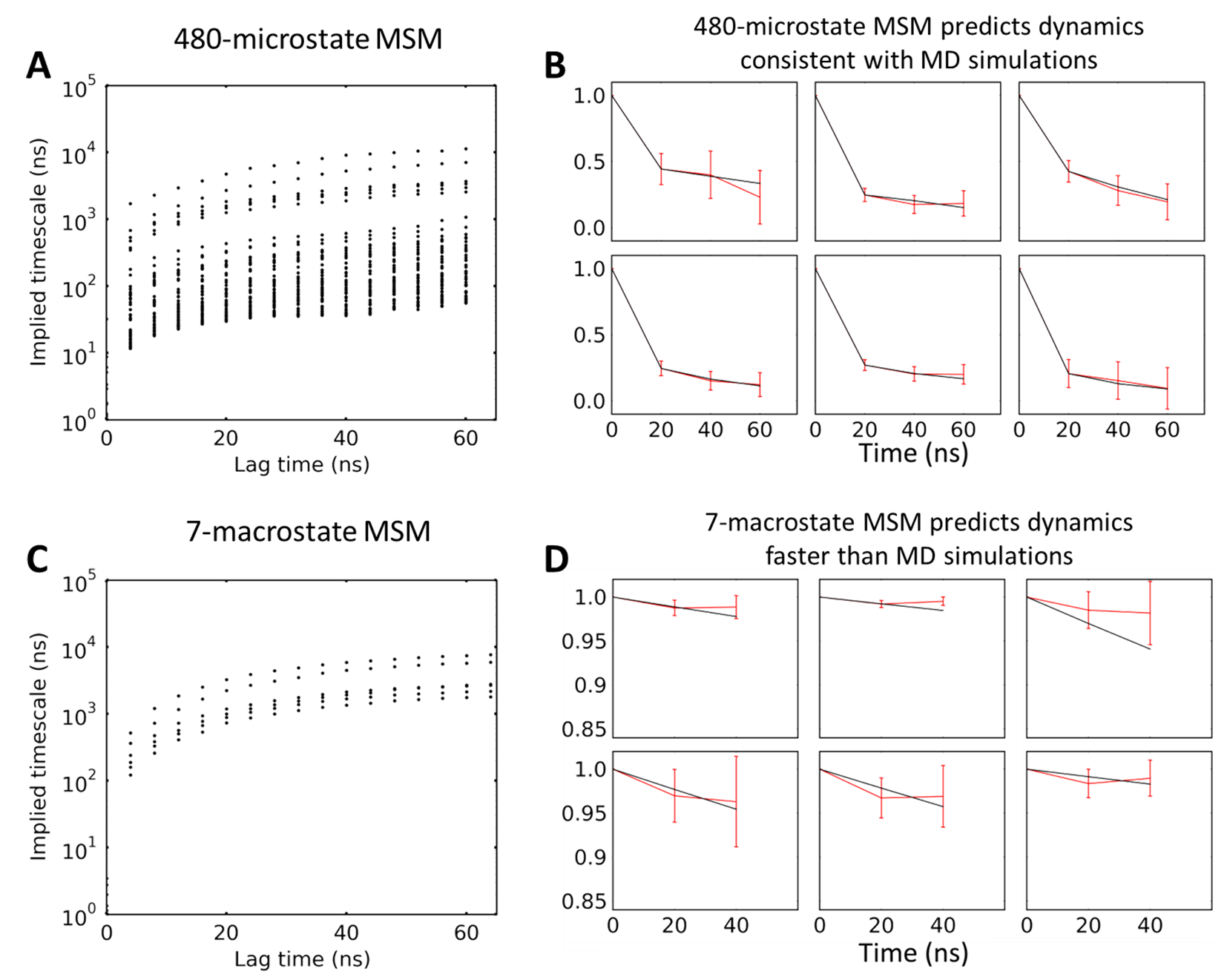

Supplement: S2 Fig — (A) Implied timescale of microstates. (B) Residence probability tests of randomly selected representative microstates. The red curves indicate probability obtained from MD simulations and the black curves indicate probability predicted by MSM. (C) Implied timescale of macrostates. (D) Residence probability tests of macrostates. The coloring scheme is the same with (B). (TIF) [file pcbi.1004404.s002.tif]

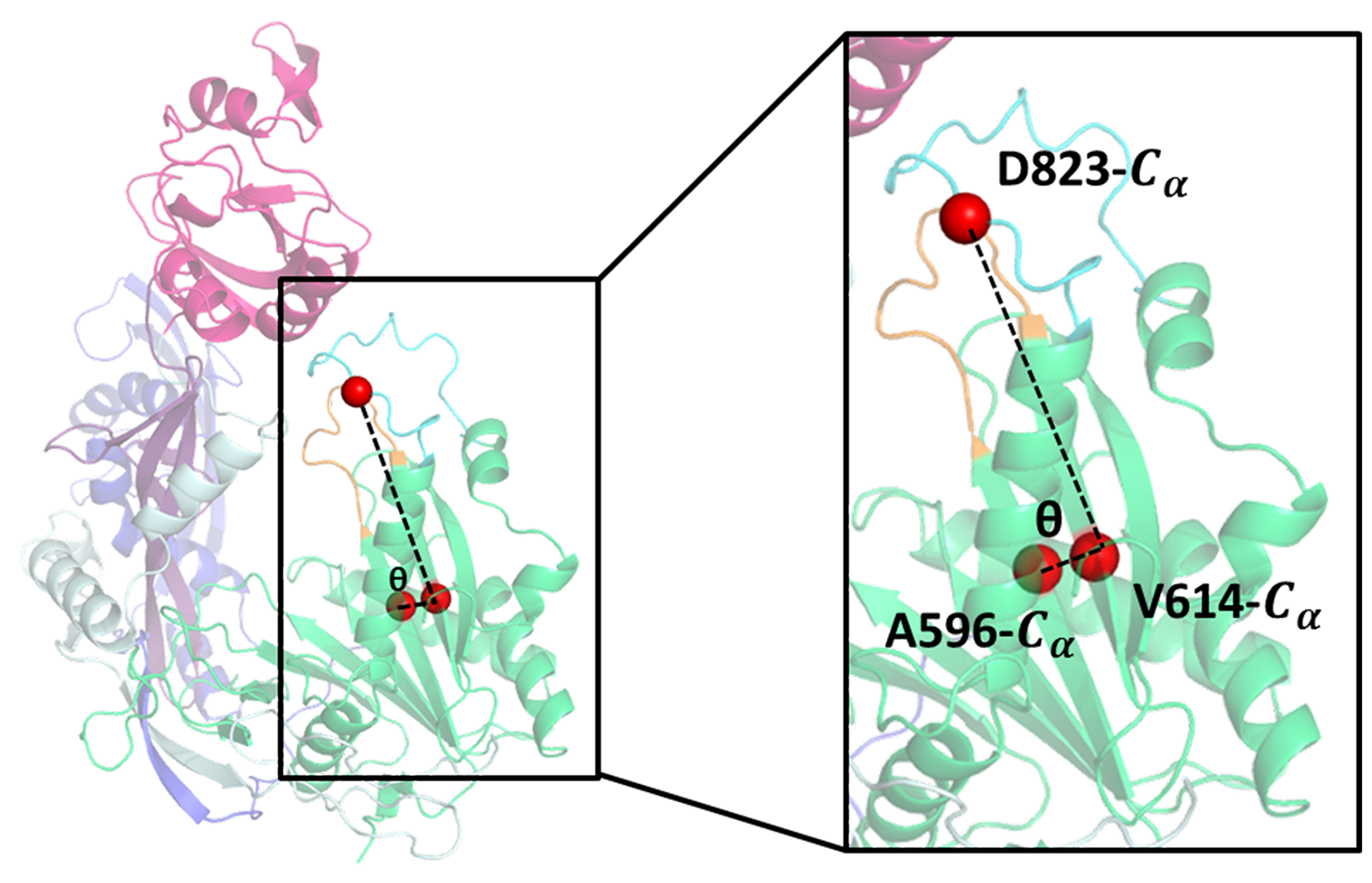

Supplement: S3 Fig — This angle describes the position of the Cα atom in D823, a residue on major PIWI loop which frequently participates in the interaction with PAZ. It thereby indicates the conformation of the major PIWI loop. When the angle is small, the major loop flips towards PAZ and easily forms hydrogen bonding network; when this angle is large, the major loop flips away from PAZ and diminishes PAZ-PIWI loops interactions. MID domain is not shown for a clear view on the angle. (TIF) [file pcbi.1004404.s003.tif]

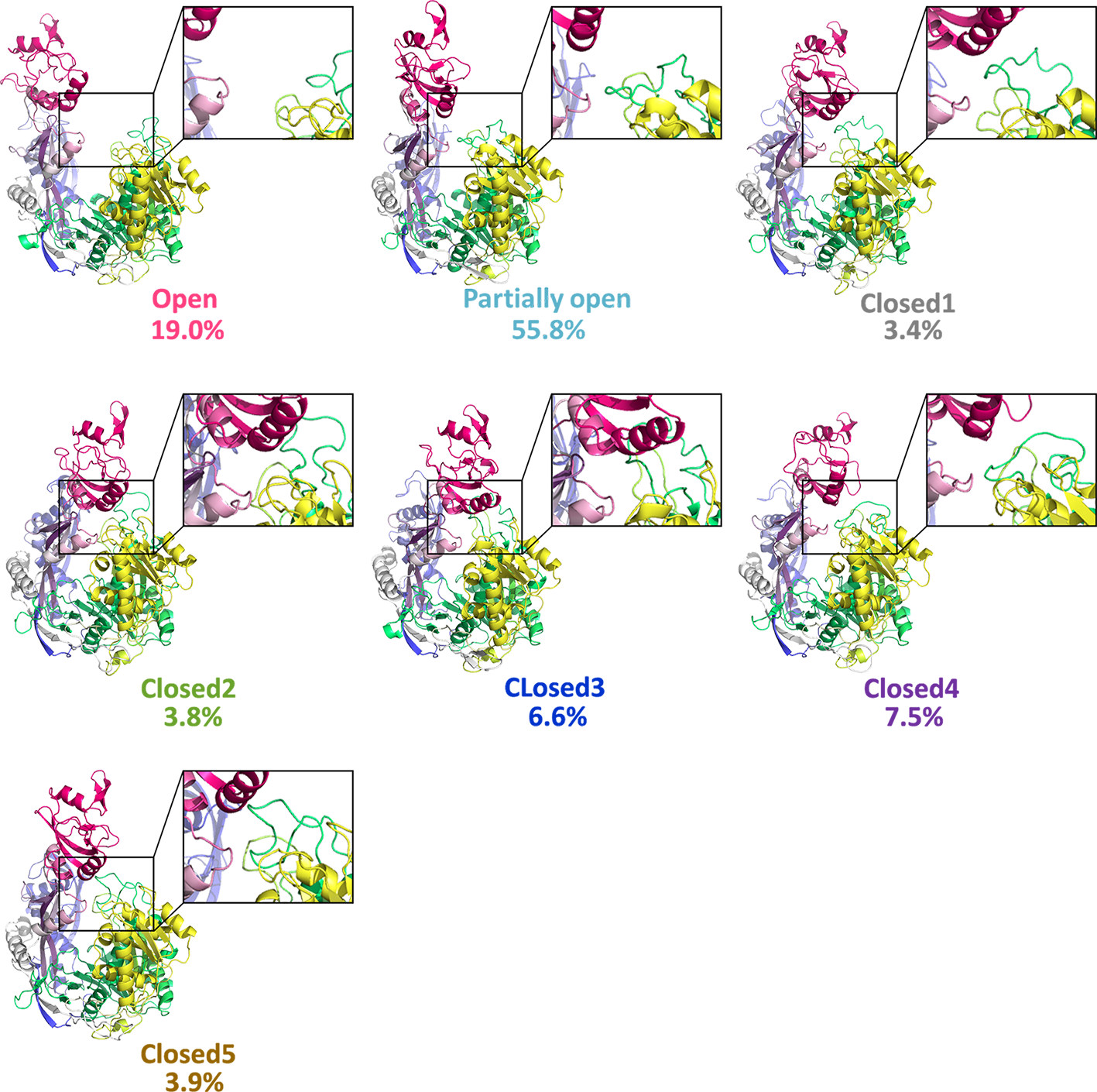

Supplement: S4 Fig — The structures of the PAZ domain and the PIWI loops are highlighted in the inset panels. Their equilibrium populations predicted from our MSM are also shown. (TIF) [file pcbi.1004404.s004.tif]

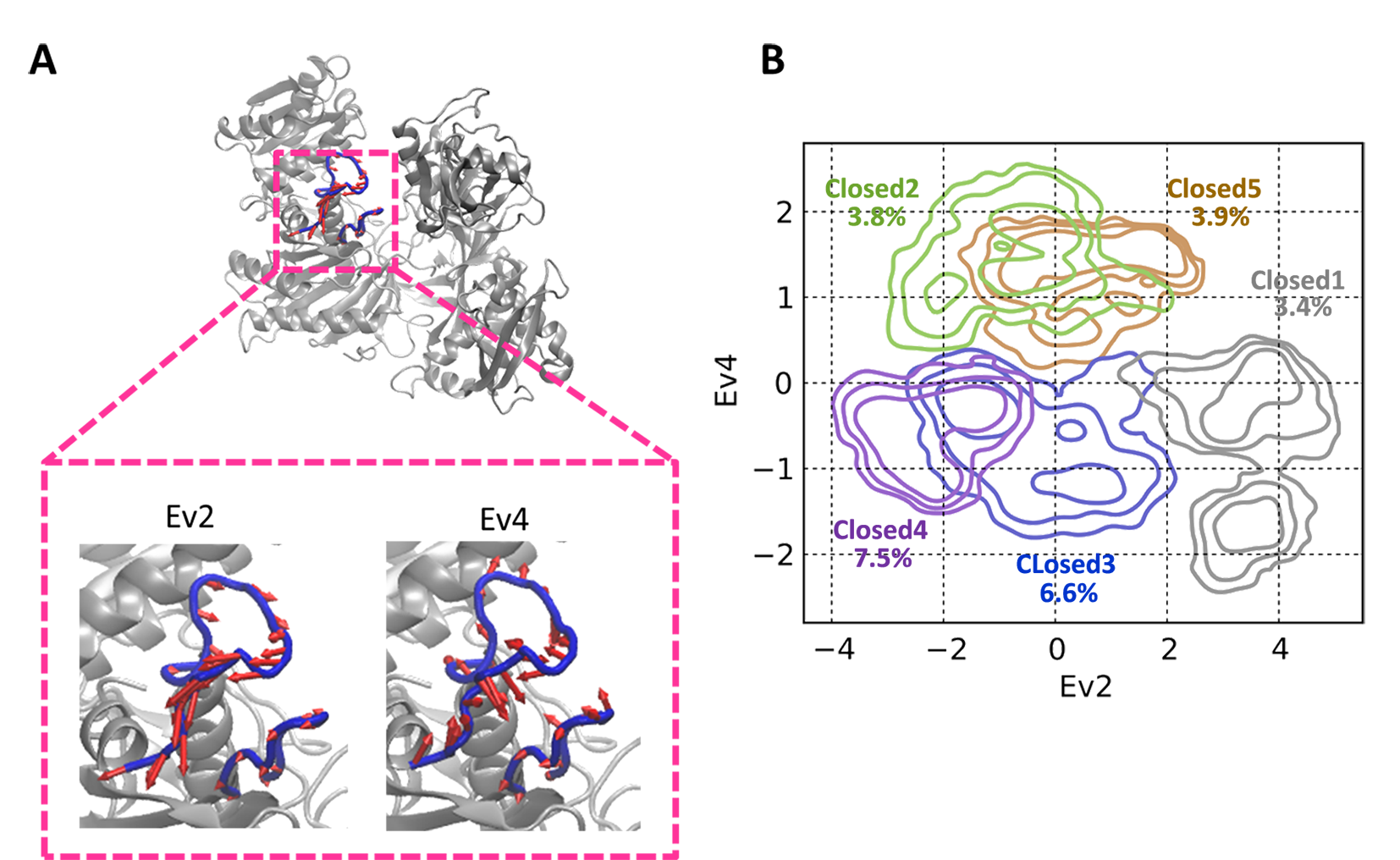

Supplement: S5 Fig — (A) The 2nd and 4th eigenvectors from the PCA. PIWI loops are colored in blue and red arrows mark the direction of the corresponding eigenvector. (B) Projection of the five closed states on the plane of the 2nd and 4th eigenvectors. The 2nd and 4th eigenvectors were chosen for projection because they can clearly separate the closed macrostates from each other. (TIF) [file pcbi.1004404.s005.tif]

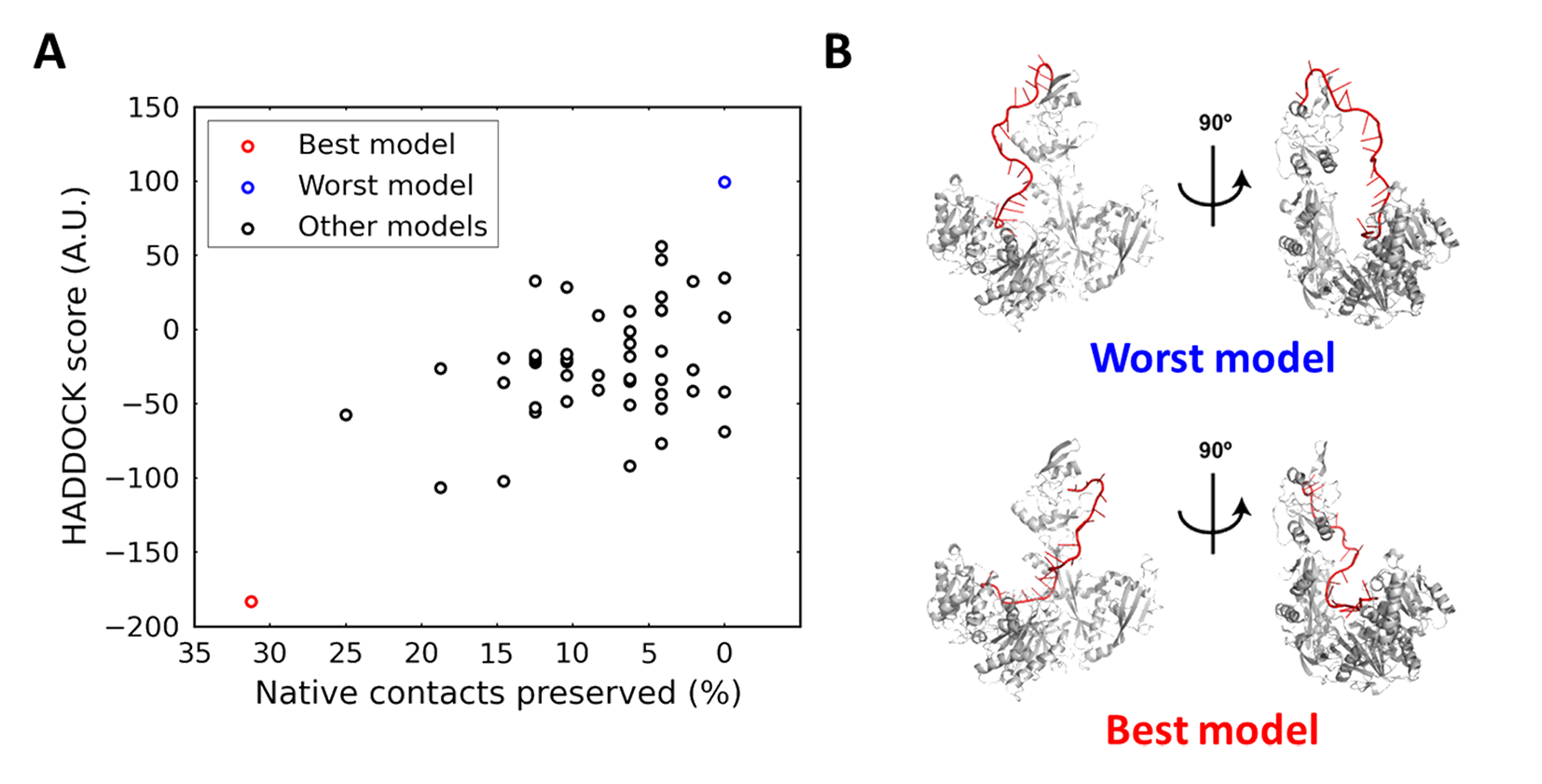

Supplement: S6 Fig — (A) Projection of 50 binary models from one protein-RNA docking simulation on their HADDOCK scores (vertical axis) and the fraction of native contacts preserved of the models (horizontal axis). (B) Structures of the worst (top) and the best (bottom) docking binary models from the 50 models presented in (A). (TIF) [file pcbi.1004404.s006.tif]

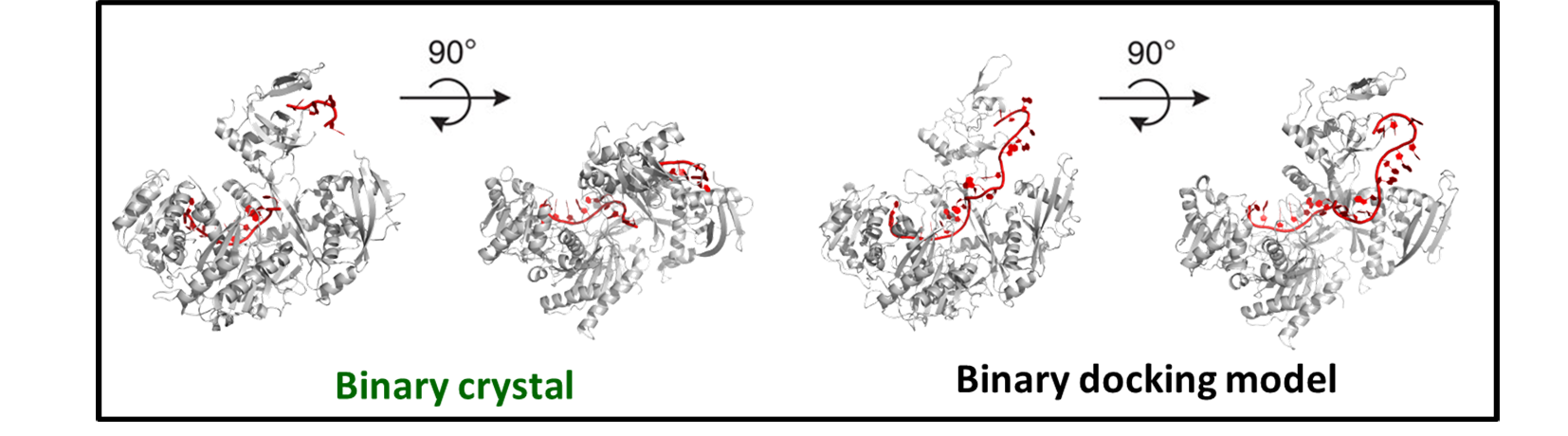

Supplement: S7 Fig — (TIF) [file pcbi.1004404.s007.tif]

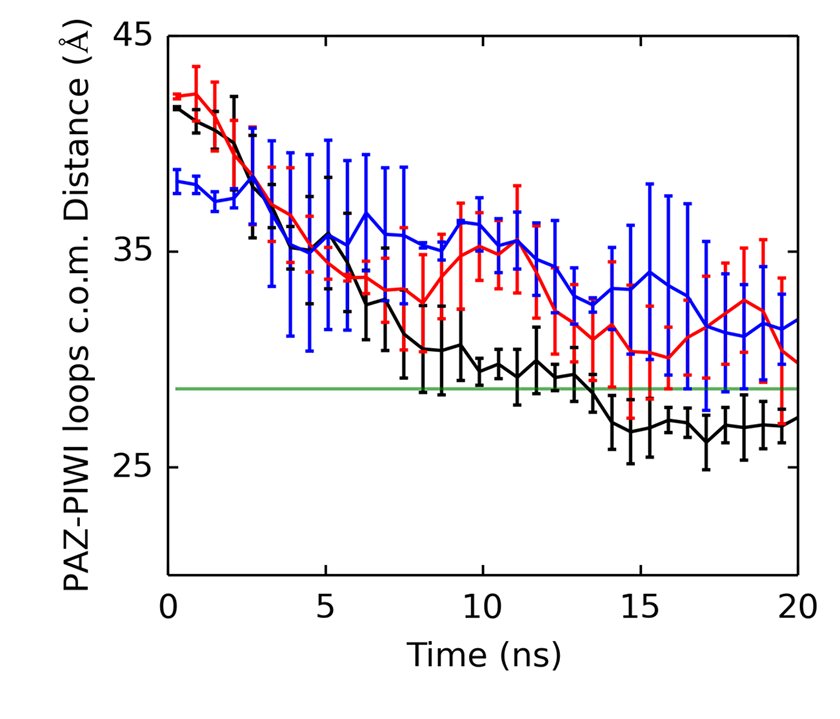

Supplement: S8 Fig — Each curve shows the average and the fluctuation of three individual trajectories from the same docked structure. The green line marks the PAZ-PIWI loops c.o.m. distance of the partially open crystal hAgo2 structure. (TIF) [file pcbi.1004404.s008.tif]

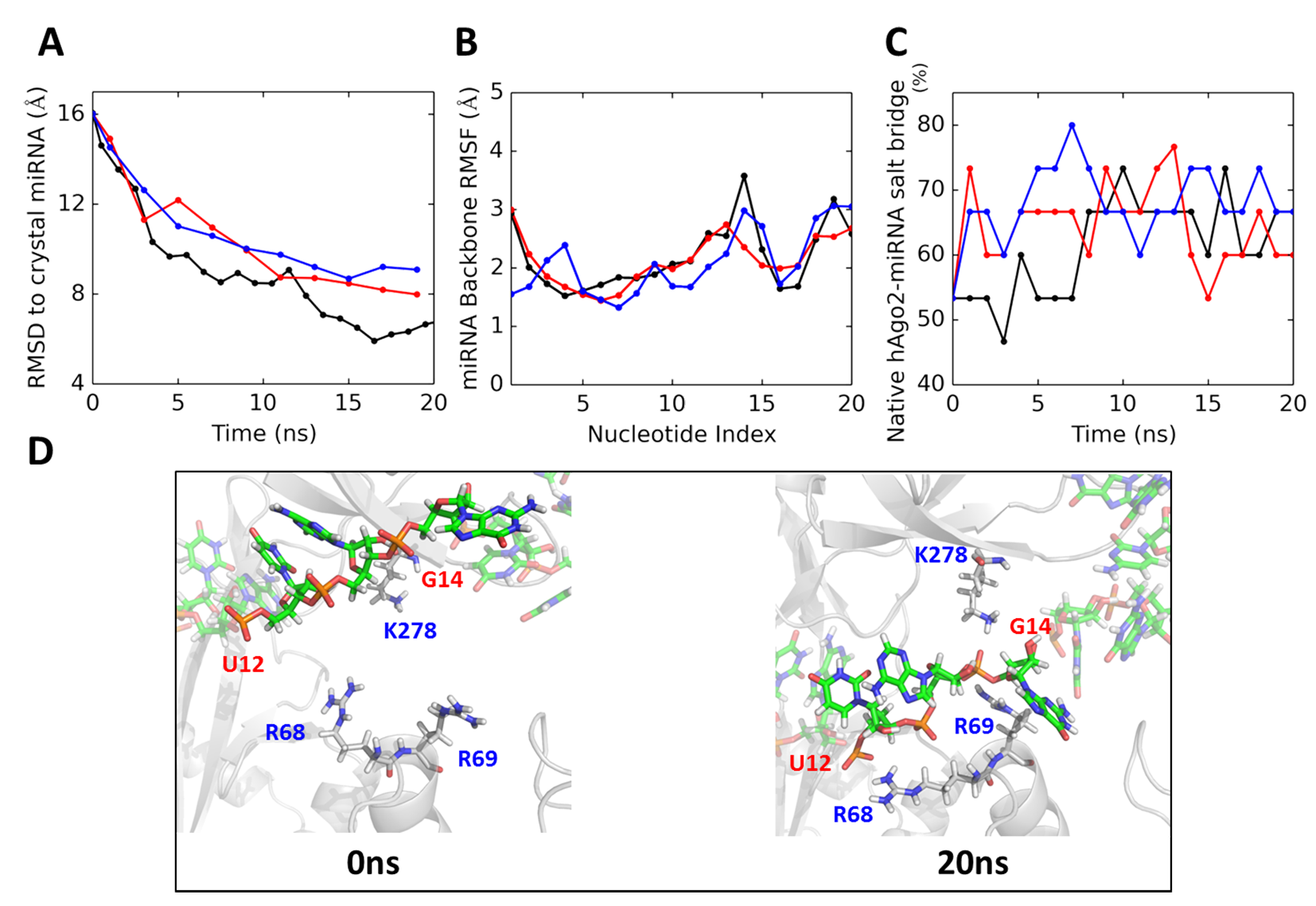

Supplement: S9 Fig — The results of three MD simulations from a successfully docked hAgo2-miRNA structure are shown. (B) Backbone Root Mean Square Fluctuations (RMSFs) of individual nucleotide in the miRNA. (C) Fraction of the native hAgo2-miRNA salt bridges formed as a function of simulation time. Results from the same three MD simulations as in (A) are shown. Native salt bridges are defined as those found in the binary hAgo2-miRNA crystal structure. (D) Representative snapshots from a MD simulation indicate the formation of the salt bridges: R68-U12, R69-G14 and K278-G14. The snapshots are selected from the MD simulation shown in black in part (A). (TIF) [file pcbi.1004404.s009.tif]

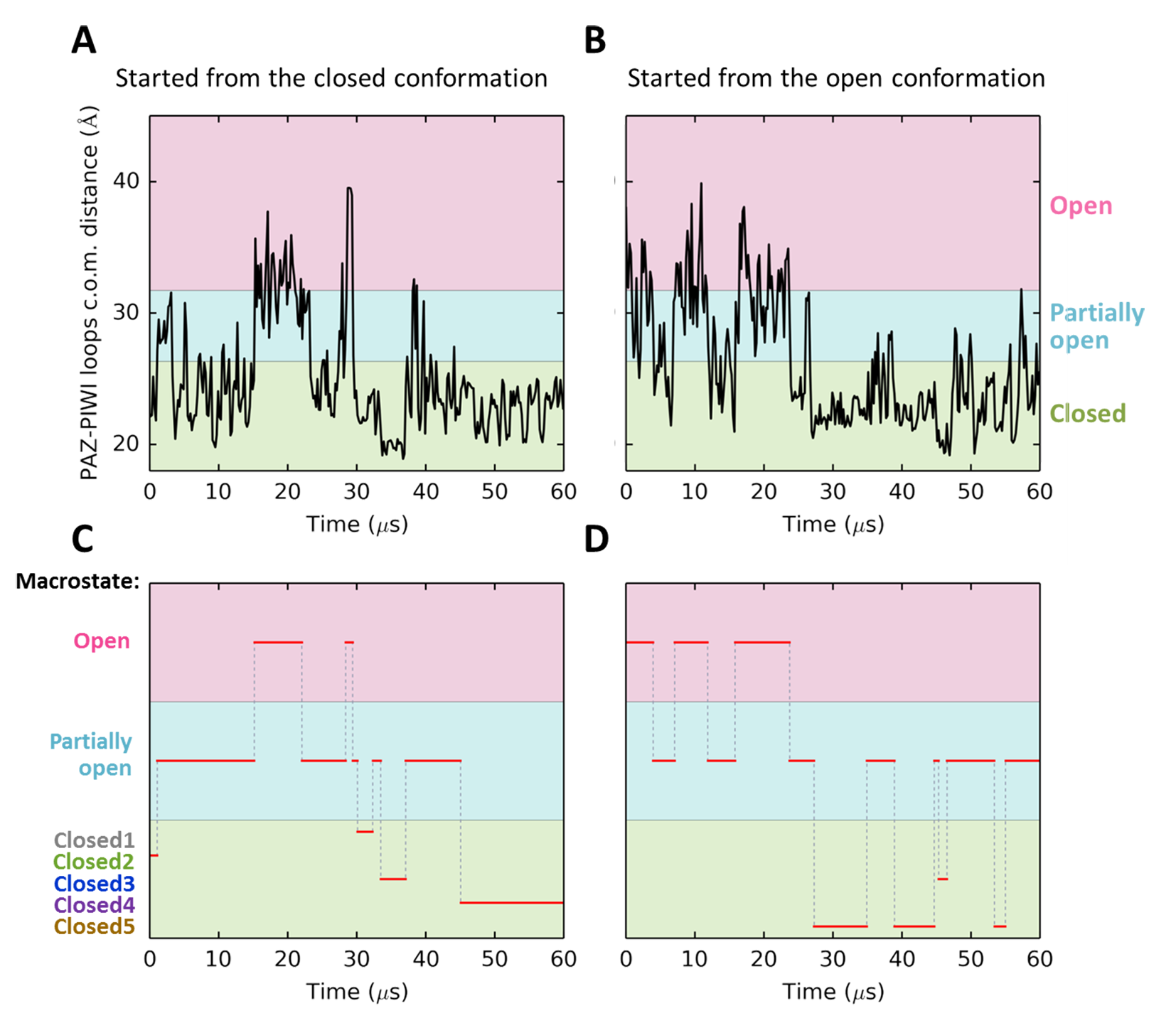

Supplement: S10 Fig — (A) PAZ-PIWI loops c.o.m. distance as a function of time in an MSM-predicted 60μs segment of trajectory. This trajectory is initiated from a closed conformation (Closed2 state). To obtain this trajectory, we sample the transition probability matrix of the validated 480-microstate MSM. In each time-step (20ns as the lag time in our MSM), the next microstate that the system will visit is determined by a randomly selected microstate according to the corresponding transition probability in the MSM. A random conformation from this microstate is then selected to compute the PAZ-PIWI loops c.o.m. distance. (B) The same as (A) except that the trajectory is started from the open conformation. (C) The same as (A) except that the macrostate ID as a function of time is displayed. To obtain the macrostate ID, we simply map the microstate the system visits to its corresponding macrostate. (D) The same as (B) except that the macrostate ID as a function of time is displayed. (TIF) [file pcbi.1004404.s010.tif]

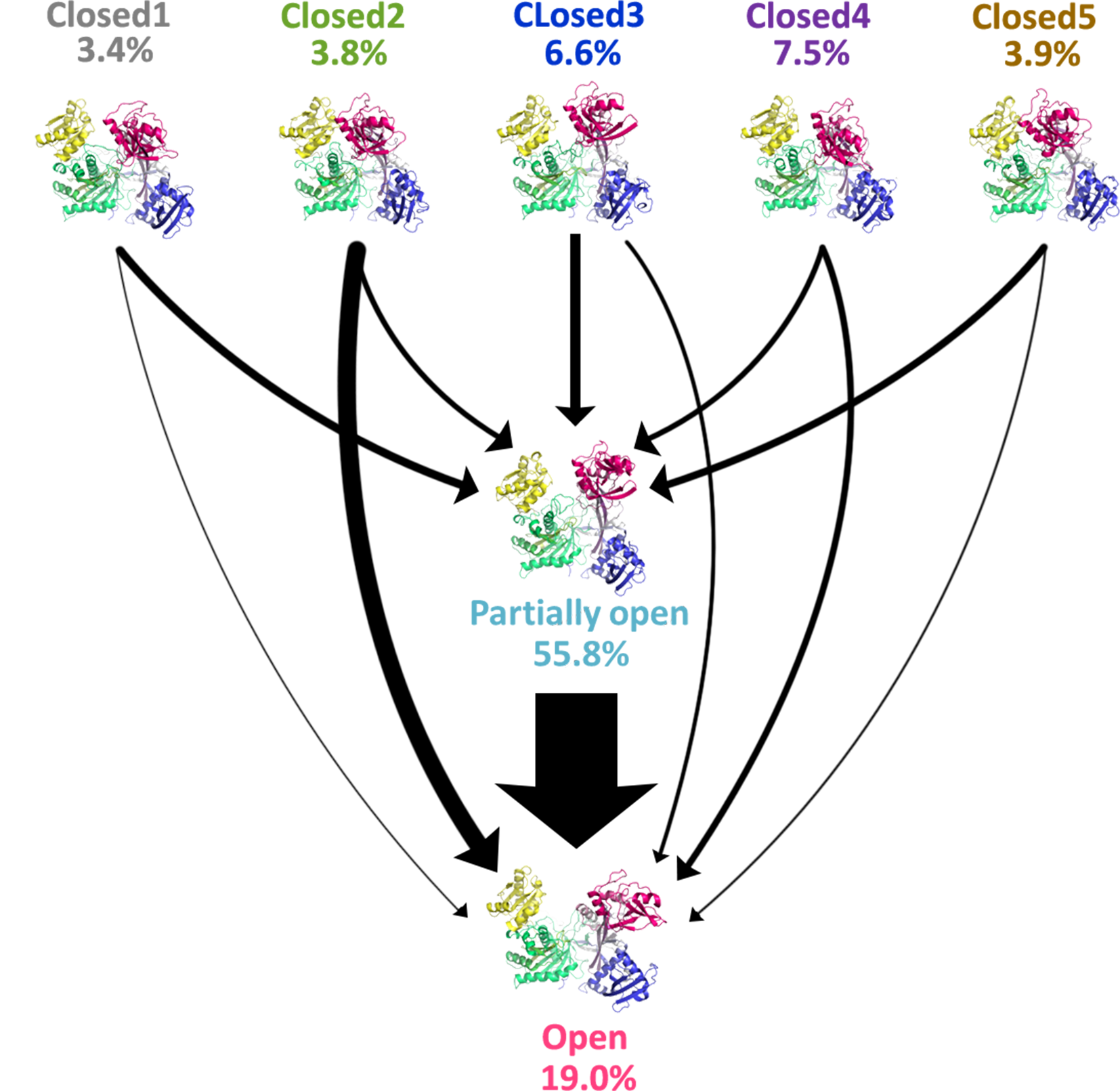

Supplement: S11 Fig — The flux is calculated using a greedy backtracking algorithm from the validated 480-microstate MSM[59] and (Weinan et al, J. Stat. Phy., 2006, 503–523). By applying the Transition Path Theory (Weinan et al, J. Stat. Phy., 2006, 503–523) to the 480-microstate MSM, we identify over one thousand pathways based on the microstates that can be further combined into ten pathways from the closed macrostates to the open macrostate. For each state a representative structure is displayed together with the MSM-predicted equilibrium population. The size of the arrows is proportional to the interstate flux. (TIF) [file pcbi.1004404.s011.tif]

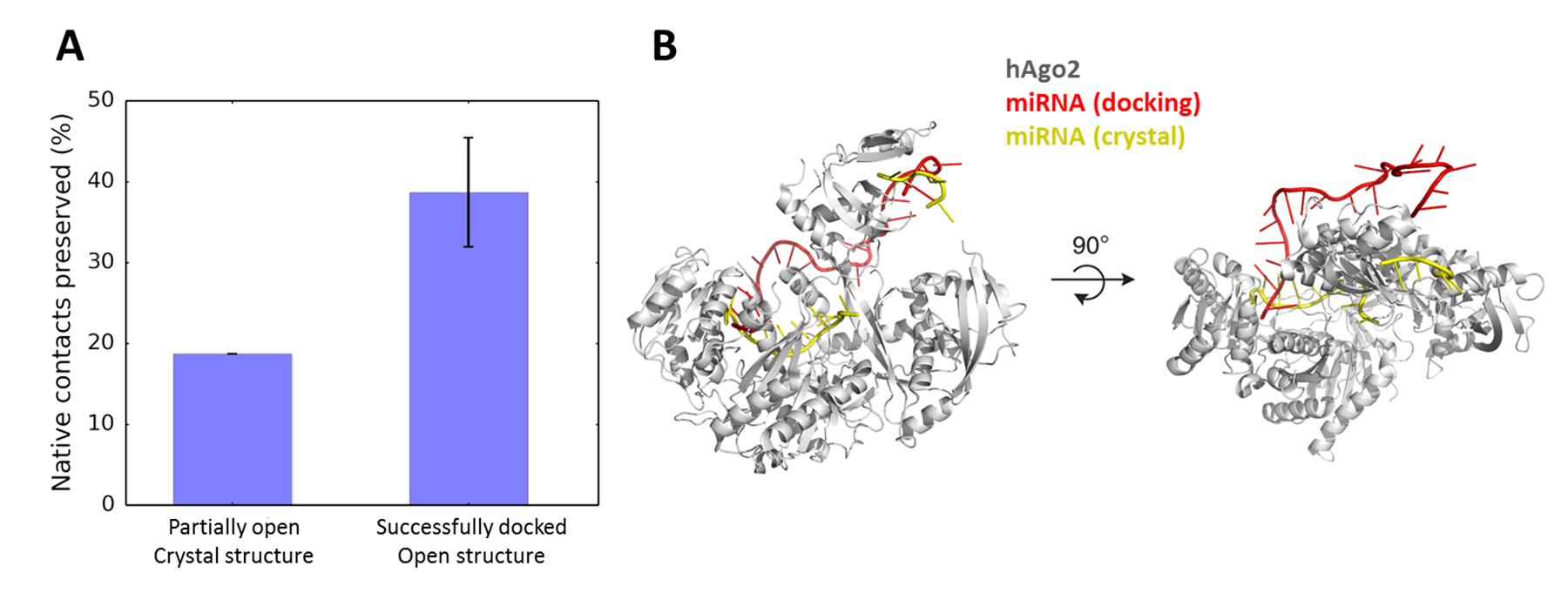

Supplement: S12 Fig — (A) Comparison between the preserved native contacts of the best-scoring docked crystal hAgo2 and the average native contacts of all the successfully docked open conformations of hAgo2. (B) Structural comparison between the best scoring docking pose and the crystal hAgo2-miRNA complex. miRNA in the docked conformation locates outside of the RNA binding groove of hAgo2. (TIF) [file pcbi.1004404.s012.tif]

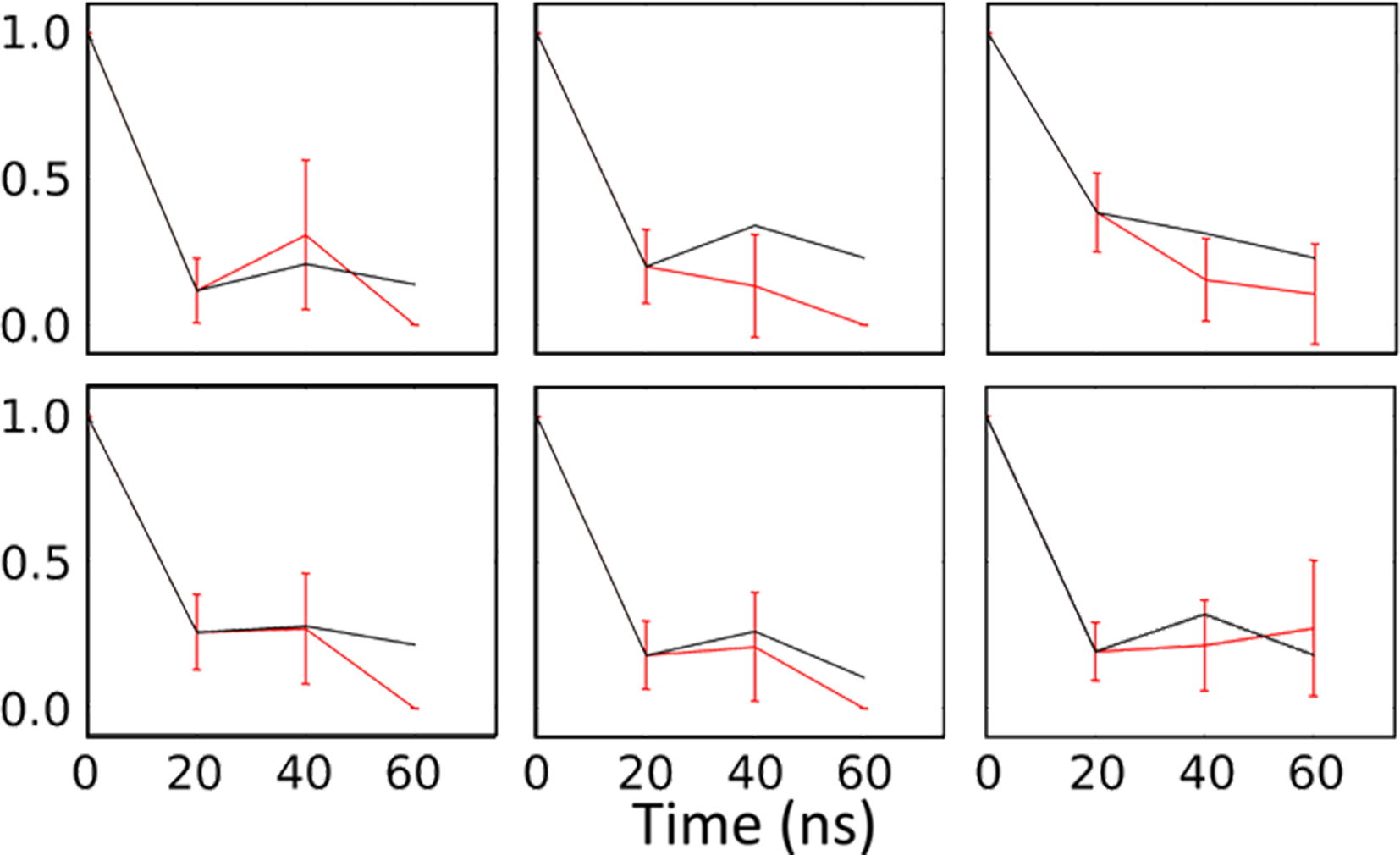

Supplement: S13 Fig — Residence probability tests of randomly selected microstates are presented. The red curves indicate probability obtained from MD simulations and the black curves indicate probability predicted by MSM. (TIF) [file pcbi.1004404.s013.tif]

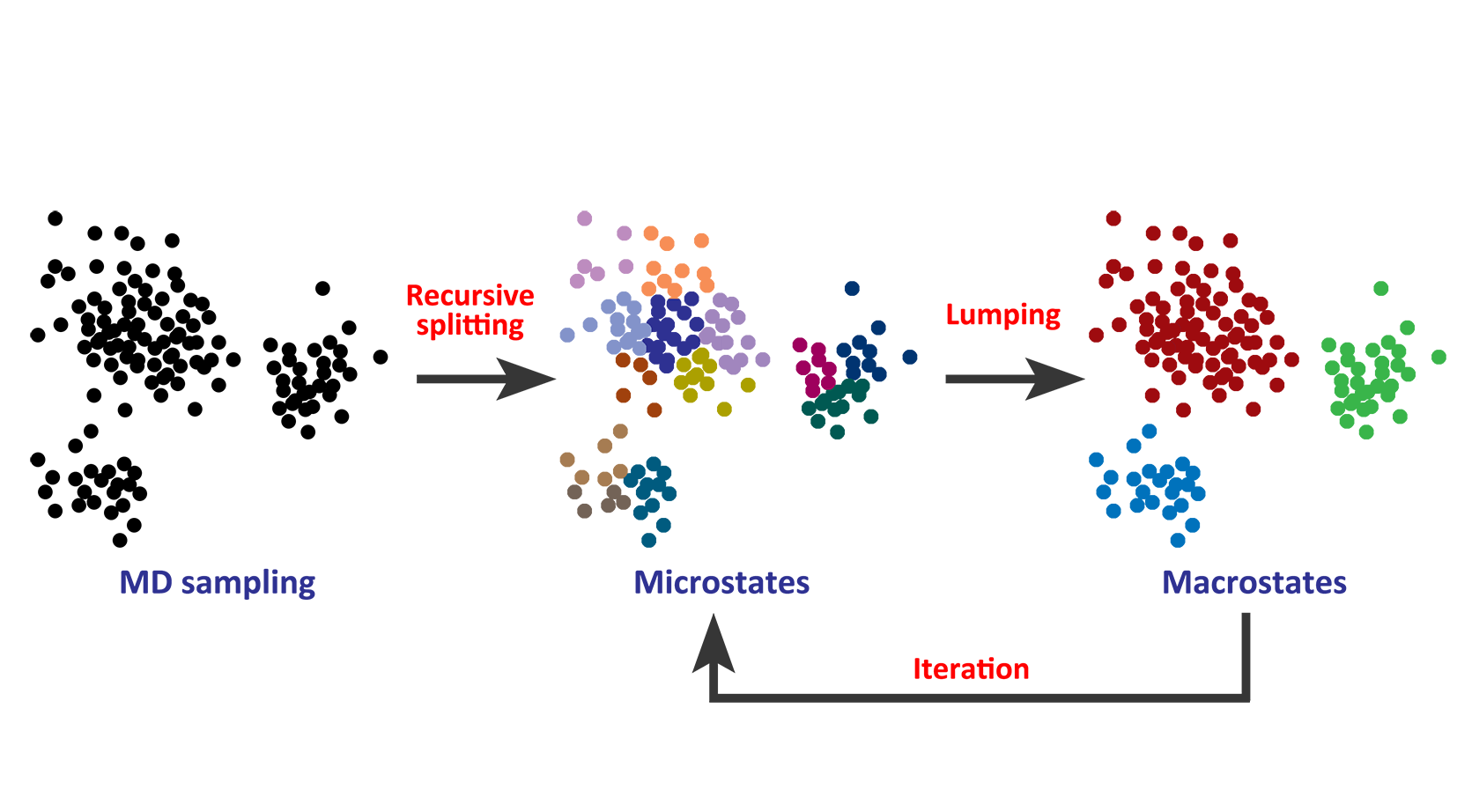

Supplement: S14 Fig — In the first step, a recursive geometric clustering is performed to divide MD conformations into microstates, until the residence times of all the microstates are below the same upper threshold. In the second step, kinetically related microstates are lumped into macrostates. Finally, multiple iterations of re-splitting and re-lumping are performed to optimize the state decomposition. (TIF) [file pcbi.1004404.s014.tif]

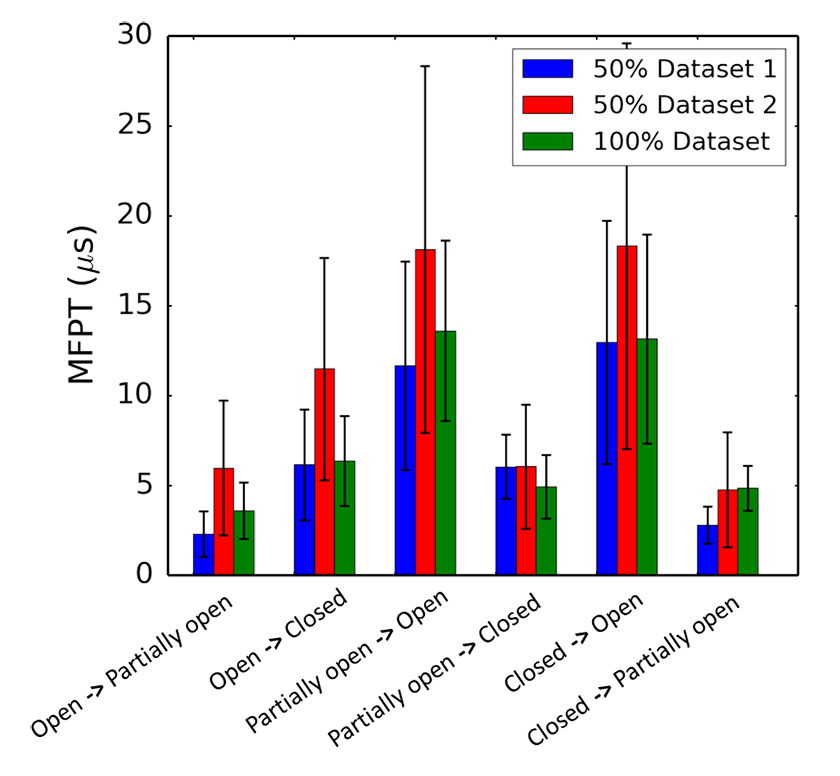

Supplement: S15 Fig — The two 50% datasets are non-overlapping subsets of the 100% dataset. The five closed states are combined and considered as one state during the calculation of MFPTs. The error bars are generated by bootstrapping N trajectories from MD dataset (N being the number of trajectories in the dataset) with replacement for N times. (TIF) [file pcbi.1004404.s015.tif]
