# Supplementary material for: Markov State Models Reveal a Two-Step Mechanism of miRNA Loading into the Human Argonaute Protein: Selective Binding followed by Structural Re-arrangement
Source: PLoS Comput Biol. 2015 Jul 16;11(7):e1004404. doi: 10.1371/journal.pcbi.1004404 (PMC4504477; doi:10.1371/journal.pcbi.1004404)
Supplement: S1 Table — Each number indicates the MFPT from the row macrostate to the column macrostate. The uncertainties were obtained from bootstrapping the MD dataset (containing 56 trajectories) with replacement for 56 times. The unit of time is μs. (PDF) [file pcbi.1004404.s016.pdf]

S1 Table

	Open	Partially open	Closed1	Closed2	Closed3	Closed4	Closed5
Open		4.0±2.1	34.4±23.7	74.2±109.3	41.1±39.6	66.2±53.4	48.6±79.2
Partially open	15.5±7.7		32.7±23.7	82.7±106.2	37.0±40.0	62.2±52.6	44.0±79.2
Closed1	19.8±11.1	6.1±7.9		84.4±107.0	43.2±40.5	68.2±53.0	50.6±78.9
Closed2	13.1±14.5	7.1±6.5	34.7±26.0		44.1±40.7	69.0±54.2	51.6±80.7
Closed3	23.5±12.3	7.8±10.4	40.5±25.7	90.5±106.9		69.5±53.3	52.0±79.8
Closed4	267.6±440.1	251.9±438.7	284.5±436.8	334.5±461.5	288.0±437.8		295.9±440.6
Closed5	17.5±7.9	1.6±0.8	34.5±23.7	84.5±106.3	38.4±40.1	63.7±52.7	
