# Supplementary material for: Markov State Models Reveal a Two-Step Mechanism of miRNA Loading into the Human Argonaute Protein: Selective Binding followed by Structural Re-arrangement
Source: PLoS Comput Biol. 2015 Jul 16;11(7):e1004404. doi: 10.1371/journal.pcbi.1004404 (PMC4504477; doi:10.1371/journal.pcbi.1004404)
Supplement: S2 Table — The restraints were selected from the closest contacts between hAgo2 and terminal nucleotides of miRNA in the crystal structure (PDB ID: 4F3T). (PDF) [file pcbi.1004404.s017.pdf]

S2 Table

hAgo2		miRNA		Distance (Å)	Error range(Å)
Residue ID	Atom name	Residue ID	Atom name		
K533	NZ	U1	OP3	2.7	±0.5
K566	NZ	U1	OP2	2.9	±0.5
H271	HE2	G20	OP1	3.1	±0.5
Y338	O	G20	O3’	2.4	±0.5
