# Supplementary material for: Markov State Models Reveal a Two-Step Mechanism of miRNA Loading into the Human Argonaute Protein: Selective Binding followed by Structural Re-arrangement
Source: PLoS Comput Biol. 2015 Jul 16;11(7):e1004404. doi: 10.1371/journal.pcbi.1004404 (PMC4504477; doi:10.1371/journal.pcbi.1004404)
Supplement: S3 Table — All the hAgo2-miRNA distances within 4Å are recorded. (PDF) [file pcbi.1004404.s018.pdf]

S3 Table

hAgo2		miRNA		Distance (Å)
Residue ID	Atom name	Residue ID	Atom name	
R812	NH2	U1	O1P	3.2
K570	NZ	U1	O2P	3.1
K566	NZ	U1	O5T	2.9
Q548	NE2	U1	O2'	2.9
C546	N	U1	O2P	3.1
Q545	NE2	U1	O5T	2.7
K533	NZ	U1	O2P	2.7
Y529	OH	U1	O1P	2.9
G524	O	U1	N3	3.4
K525	CA	U1	O4	3.7
T526	N	U1	O4	3.4
K566	CD	A2	O3'	3.3
L563	N	A2	O2'	3.7
N562	ND2	A2	N3	3.1
N551	ND2	A2	O2P	2.7
Q548	NE2	A2	O1P	2.8
T559	CG2	A2	N9	3.5
C793	CB	A3	O2'	3.2
R792	NH2	A3	O1P	3.3
N562	ND2	A3	C1'	4.0
K566	NZ	A3	O1P	2.9
Y804	OH	A4	O5'	3.5
C793	SG	A4	C4'	3.7
R792	NH1	A4	O1P	2.8
S798	OG	G5	O1P	2.6
Y804	OH	G5	O2P	2.5
Q757	OE1	G5	N2	2.5
I365	CG2	U6	O2	3.3
R761	N	U6	O1P	2.8
Q757	O	U6	O4'	2.9
K709	NZ	U6	O2P	3.2
A221	O	G7	O2'	2.8
I365	CG1	G7	C5	3.7
R761	NE	G7	O2P	2.8
R351	NH2	C8	O3'	3.9
A221	O	C8	C4'	3.3
R761	NH2	C8	O2P	2.4

T222	OG1	C8	O2'	3.1
R710	NE	U9	C5	3.1
R351	NH2	U9	O1P	2.2
R710	NH2	U10	O1P	2.5
R351	NH1	U10	C5'	3.7
R635	NH1	U10	C5	3.1
Y311	OH	G19	O1P	3.1
F294	CE2	G20	C5	3.2
Y311	OH	G20	O2P	2.2
H316	NE2	G20	O2P	3.4
H271	NE2	G20	O1P	3.1
