# Supplementary material for: Markov State Models Reveal a Two-Step Mechanism of miRNA Loading into the Human Argonaute Protein: Selective Binding followed by Structural Re-arrangement
Source: PLoS Comput Biol. 2015 Jul 16;11(7):e1004404. doi: 10.1371/journal.pcbi.1004404 (PMC4504477; doi:10.1371/journal.pcbi.1004404)
Supplement: S1 Text — (PDF) [file pcbi.1004404.s019.pdf]

S1 Text. Comparison between apo hAgo2 dynamics and kinetics of hAgo2-miRNA collision
We have compared the kinetics of the open-closed transition (a unimolecular event) and that of hAgo2-miRNA collision (a bimolecular event). The units of the rate constants in these two events are different because different numbers of molecules are involved. Therefore, directly comparing the two rate constants is not feasible. Moreover, it has been recently reported that both protein dynamics and the ligand concentration can influence the recognition mechanism[30]. We therefore have compared the rates, instead of the rate constants, of the two events to demonstrate that in physiological condition the selective binding of open hAgo2 by miRNA is possible. An estimated transition rate ($\nu_{closed-open}$) from a closed hAgo2 to an open conformation is obtained as:

$$\nu_{closed-open} = \frac{1}{t_{closed-open}}[P] = \frac{1}{10\mu s} \times 500nM = 5 \times 10^{-2} Ms^{-1} \qquad \text{(S1)}$$

where $t_{closed-open}$ denotes the mean first passage time (MFPT) from a closed state to the open state derived from our MSM (see Table S3). Although MFPTs from different closed states to the open state show certain discrepancy, the majority of them are at tens of μs. Therefore, we choose 10 μs as an estimated value here to represent the correct order of magnitude. [P] is the experimental hAgo2 concentration[35]. Recent experiments showed that single strand siRNA collides with hAgo2 at a rate constant of around $1 \times 10^8 M^{-1}s^{-1}$[35]. Thus the hAgo2-miRNA collision rate ($\nu_{collision}$) is:

$$\nu_{collision} = k_{collision}[L][P] = 1 \times 10^8 M^{-1}s^{-1} \times 20 \times 10^{-9} M \times 500nM = 1 \times 10^{-6} Ms^{-1} \qquad \text{(S2)}$$

where $k_{collision}$ is the hAgo2-RNA collision rate constant, [L] is the experimental RNA concentration[35] and [P] is the experimental hAgo2 concentration[35].

The MSM-predicted transitioning rate to the open state is significantly faster than the collision rate, suggesting that the apo hAgo2 always has sufficient time to reach open state before it encounters miRNA.
