# Supplementary material for: Markov State Models Reveal a Two-Step Mechanism of miRNA Loading into the Human Argonaute Protein: Selective Binding followed by Structural Re-arrangement
Source: PLoS Comput Biol. 2015 Jul 16;11(7):e1004404. doi: 10.1371/journal.pcbi.1004404 (PMC4504477; doi:10.1371/journal.pcbi.1004404)
Supplement: S2 Text — (PDF) [file pcbi.1004404.s020.pdf]

S2 Text. PAZ-PIWI loops interaction could enhance the fidelity of mRNA target recognition

Interestingly, we find the interaction between PAZ and PIWI loops exist in the binary hAgo2-miRNA complex as well as in apo hAgo2. As shown in Fig. S2TA&B, both PIWI loops can form extensive interactions with PAZ through salt bridges and hydrogen bonds. This PAZ-PIWI loops interaction could play an important role during mRNA recognition by hAgo2-miRNA complex. In the hAgo2-miRNA complex structure, the catalytic tetrad (D597-E637-D669-H807) is deeply buried below PAZ and PIWI loops and not accessible to target mRNA (see Fig. S2TA&C). Since hAgo2 is a catalytically active endonuclease, exposing the catalytic tetrad to the solvent could lead to unspecific RNA cleavage. Therefore the PAZ-PIWI loops interaction may enhance the fidelity of target mRNA recognition by avoiding accidental exposure of the catalytic tetrad.

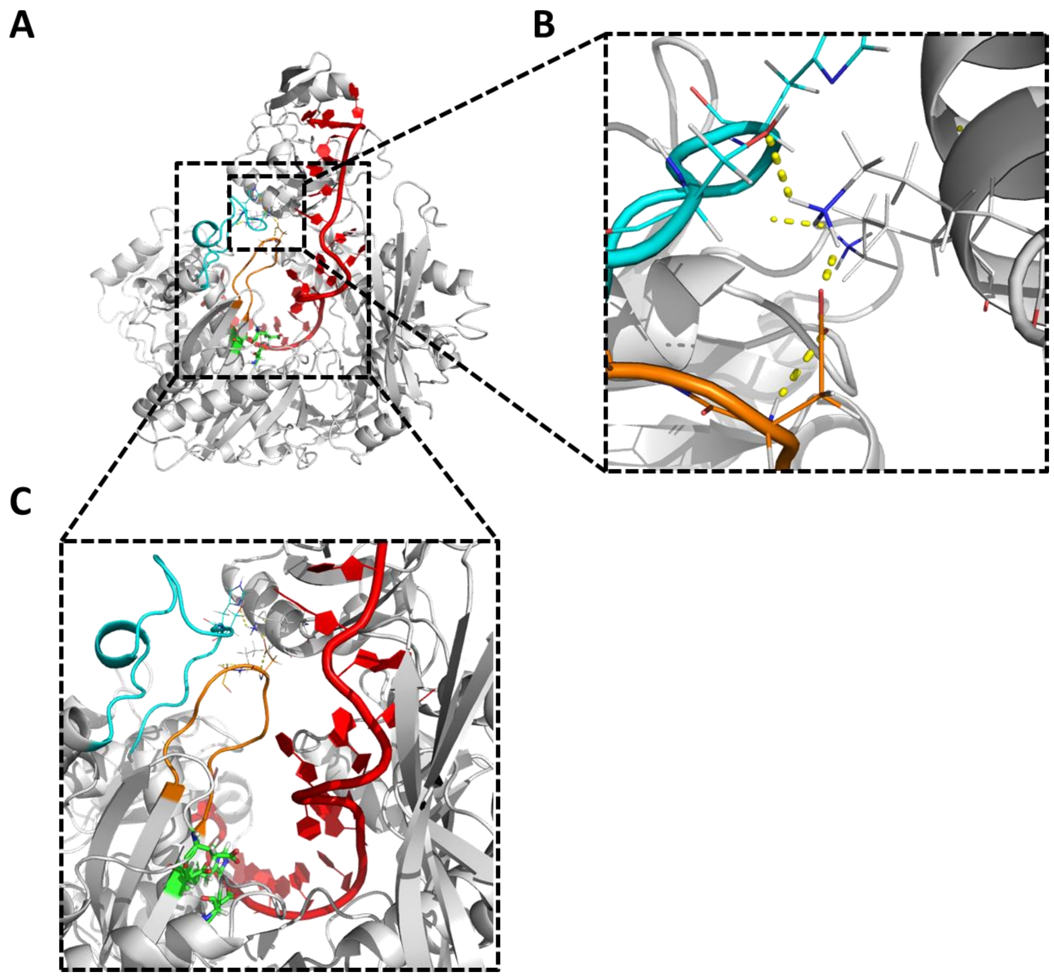

Fig. S2T (A) An hAgo2-miRNA complex conformation after 30-ns MD simulation initiated from the crystal complex. (B) Interactions between PAZ and PIWI loops (major loop in cyan and minor loop in orange). (C) Catalytic tetrad (in green) buried under PAZ and PIWI loops.
